# Camel Milk as a Functional Food: Nutritional Composition, Health‐Promoting Benefits, and Safety Considerations

**DOI:** 10.1002/fsn3.71638

**Published:** 2026-03-23

**Authors:** Gudisa Bereda, Subasini Uthirapathy, Javed Ahamad

**Affiliations:** ^1^ Department of Pharmacy Marie Stopes International Ethiopia (MSIE) Ambo Ethiopia; ^2^ Department of Pharmacology Tishk International University Erbil Iraq; ^3^ Department of Pharmacognosy Tishk International University Erbil Iraq

**Keywords:** bioactive compounds, camel milk, functional food, health benefits, nutritional composition, safety, therapeutic effects

## Abstract

Camel milk is a nutrient‐rich functional food traditionally consumed in arid regions, containing bioactive proteins, peptides, exosomes, vitamins, minerals, and insulin‐like compounds. It has therapeutic, physiological, and nutritional benefits but may carry zoonotic risks. This review evaluated both the health benefits and potential microbial and zoonotic risks of camel milk. Preclinical, clinical, and epidemiological studies from 2000 to 2025 were included. Studies reporting intervention type, sample size, duration, health outcomes, or safety considerations were included. The findings were synthesized qualitatively. This review showed that camel milk (200–500 mL/day or 10 g powder BID, 2–14 weeks) improves glycemic control in Type 2 diabetes by lowering fasting and postprandial glucose, HbA1c, and plasma insulin. In children with autism, regular consumption of camel milk enhances behavioral, social, and gastrointestinal outcomes by reducing inflammatory cytokines. Preclinical studies show that camel milk exerts cardioprotective, hepatoprotective, nephroprotective, chemopreventive, antiobesity, and antiaging effects through antioxidant and anti‐inflammatory mechanisms. Additionally, camel milk improves hemoglobin levels, supports growth, and promotes beneficial gut microbiota. It is derived probiotics and exosomes provide antimicrobial, anticancer, and immunomodulatory effects, and they are safe and well‐tolerated in children. However, concerns related to raw camel milk consumption, including microbial contamination and zoonotic transmission, warrant careful consideration. Camel milk provides nutritional and therapeutic benefits, enhanced by probiotics and bioactive derivatives. However, unpasteurized milk may carry zoonotic pathogens and must be handled carefully.

## Graphical abstract

Camel milk contains low lactose, unique casein proteins, high vitamin contents, and diverse bioactive compounds that support metabolic health, immune function, organ protection, gut health, physical development, and brain function. Its benefits are mediated through antioxidant, anti‐inflammatory, insulin‐like effects, and immunomodulatory mechanisms. Pasteurized camel milk is safe for consumption, whereas raw camel milk may pose microbial and zoonotic risks if not properly handled.

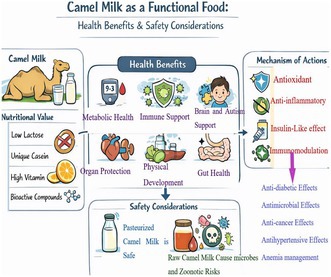

## 1 Introduction

A preprint version of this manuscript has been previously published by Bereda (2024) on the Research Square preprint server with https://doi.org/10.20944/preprints202411.1552.v1. The camel is a desert mammal belonging to the Camelidae family and the Artiodactyla suborder. The Bactrian two‐humped camel ( *Camelus bactrianus* ) and the Arabian or dromedary one‐humped camel ( *Camelus dromedarius* ) are the two primary varieties of camels (Swelum et al. 2021). Increasingly, people are raising camels for both milk and meat (Ramadan and Inoue‐Murayama 2017). Both breeds produce milk rich in minerals, vitamins, and unsaturated fats, with some macronutrient variations depending on location (Mihic et al. 2016). According to the Food and Agriculture Organization (FAO), the global camel population is estimated at 19 million, with 15 million in Africa and 4 million in Asia (Galali and Al‐Dmoor 2019). Camel milk, often referred to as the “white gold of the desert,” is dark white with a harsh, salty flavor and pungent odor, varying with breed, diet, health, and water intake (Sabry et al. 2021; Mal et al. 2006). At 2°C, camel milk maintains quality for up to 12 days, compared with 2–3 days for cow milk (Mal et al. 2006); raw camel milk lasts 8–9 h at 37°C and over a week at 4°C–6°C (Singh 2017).

Consumed for centuries in arid and semi‐arid regions, camel milk is a nutrient‐dense functional food rich in bioactive proteins, peptides, exosomes, vitamins, minerals, and insulin‐like compounds (Kumar et al. 2016a, 2016b). These components collectively support its therapeutic, physiological, and nutritional properties. Camel milk has demonstrated potential in managing metabolic disorders such as Type 2 diabetes, improving glycemic control, lipid profiles, and insulin sensitivity (Zheng et al. 2021). Evidence also supports its immunomodulatory and anti‐inflammatory effects, with benefits in autism spectrum disorders, cardiovascular, hepatic, and renal diseases, as well as oxidative stress‐related conditions (Nguyen et al. 2016). Beyond therapeutic effects, camel milk provides physiological benefits, including improved growth, enhanced hemoglobin levels, and reduced prevalence of stunting and underweight in children from pastoralist communities (Sakandar et al. 2018). Camel milk contains bioactive components such as probiotics, fermented products, casein hydrolysates, whey proteins, and exosomes (Kumar et al. 2016a). These components have demonstrated antimicrobial, anticancer, and gut‐modulating activities in preclinical and clinical studies. Camel milk is generally safe and well‐tolerated, even in children with cow milk protein allergy (Navarrete‐Rodríguez et al. 2018); however, consumption of raw milk requires caution due to potential zoonotic and prion risks.

Camel milk has a long history of traditional use, yet its multidimensional health benefits, therapeutic, physiological, and nutritional, have only recently been systematically investigated. While numerous preclinical and clinical studies exist, a comprehensive synthesis integrating mechanistic insights and clinical evidence is lacking. This review was therefore undertaken to consolidate current knowledge on camel milk as a functional food, highlighting its applications in modern healthcare, disease prevention, and dietary interventions. Additionally, understanding its safety profile, including potential risks associated with raw milk, is essential for guiding safe consumption and promoting evidence‐based recommendations. It also addresses safety considerations to ensure safe consumption.

## 2 Methods

### 2.1 Study Design

This study adopts a narrative review design, synthesizing existing scientific literature on the medicinal, physiological, and nutritional benefits of camel milk. Both preclinical and clinical evidence are integrated to provide a comprehensive overview of the health‐promoting properties of camel milk and its bioactive components. The review also addresses potential safety considerations, including zoonotic risks associated with the consumption of raw camel milk.

### 2.2 Research Question

The primary research question guiding this review is:

1. What are the medicinal, physiological, and nutritional benefits of camel milk, and how do its bioactive components contribute to human health?
2. What is its role in disease prevention and therapeutic management?
3. What safety considerations are associated with camel milk consumption, including zoonotic risks and the need for proper processing or pasteurization?

By addressing these questions, the review highlights camel milk's potential as a functional food with significant health‐promoting properties. It also emphasizes the zoonotic risks associated with consuming raw camel milk.

### 2.3 Eligibility Criteria

Studies were included if they specifically investigated the medicinal, physiological, or nutritional properties of camel milk, including dromedary, Bactrian, and hybrid milk, as well as its bioactive components, using human or animal models. Eligible studies comprised human clinical trials (randomized controlled trials, cohort, or case–control studies), preclinical experiments (animal or in vitro models), and observational research that provided relevant data on camel milk consumption or exposure outcomes. Only full text, peer‐reviewed articles published in English between 2000 and 2025 were considered to ensure scientific rigor and accessibility. Exclusion criteria included studies on noncamel milk sources, review articles without primary data, editorials, conference abstracts lacking full results, non‐English publications, duplicates, and studies with incomplete, inconclusive, or low‐quality data. Additionally, studies reporting microbial contamination were considered to assess the potential safety risks of camel milk.

### 2.4 Search Strategy and Keywords

A comprehensive literature search was conducted across PubMed, Scopus, Web of Science, and Google Scholar to identify studies on camel milk and its health benefits. Keywords included terms for camel milk (“Camel milk” OR “ *Camelus dromedarius* milk” OR “Bactrian camel milk” OR “camel dairy” OR “camel milk products”) combined with health‐related terms (“health benefits” OR “therapeutic potential” OR “bioactive compounds” OR “functional food”). Additional terms captured specific effects on immunity, metabolic health, gastrointestinal health, and antioxidant or antimicrobial activity. Safety considerations were included using keywords such as (“safety” OR “microbial contamination” OR “pathogens” OR “foodborne infections” OR “zoonotic risk”).

### 2.5 Data Extraction and Tool

Data were systematically extracted using a structured Microsoft Excel sheet. Extracted information included author(s) and year, study type, bioactive components, reported health benefits, study population, mechanisms of action, study limitations, and any reported safety or microbial risks. This approach ensured comprehensive and consistent coverage of relevant findings.

### 2.6 Data Analysis and Synthesis

Collected data were analyzed using a thematic synthesis approach, with studies categorized by health benefits. A comparative analysis was conducted to assess consistencies and variations across studies. Findings were presented in descriptive tables and summarized qualitatively. Safety considerations were also addressed.

## 3 Camel Breed

Camel populations can be found in large parts of the arid regions of Africa, Asia, and Australia (Alhajeri et al. 2021). The family of Camelidae comprised two main types (large and small camelids) distributed into three genera: Camelus, Lama, and Vicugna (Table 1). The small camelids originate from the Andes Mountains of South America, including two domestic species (lama and alpaca) and two wild species (guanaco in genus lama, and vicuna in genus Vicugna). The large camelids are represented by two domesticated species, the one‐humped camel (dromedary) and the two‐humped camel (Bactrian), the first living in the hot arid lands from North Africa and the eastern part of Asia, the second in the cold steppes and deserts in Central Asia (Faye 2015).

**TABLE 1:** Comparison of dromedary, Bactrian, and hybrid camels.

Feature	Dromedary camel ( *Camelus dromedarius* )	Bactrian camel ( *Camelus bactrianus* )	Hybrid camel (Bactrian × Dromedary)	References
Humps	One	Two	One or two (depending on cross)	Sala (2022), Bornstein (2021)
Region/habitat	Middle East, North Africa, South Asia; hot arid deserts	Central Asia (Kazakhstan, Mongolia, China); cold deserts/steppes	Mixed regions where both species cohabit; farmed or semi‐domesticated	Sala (2022), Bornstein (2021)
Adult weight	400–600 kg	450–650 kg	400–620 kg	Köhler‐Rollefson (1991), Sala (2022)
Height at shoulder	1.8–2.0 m	1.7–2.2 m	1.7–2.1 m	Köhler‐Rollefson (1991), Sala (2022)
Milk fat content (%)	3–5	5.5–6.7	4–6	Ayadi et al. (2018), Chen et al. (2025)
Milk protein content (%)	3–4	3.5–4.5	3.3–4.2	E. S. I. El‐Agamy (2006), Seyiti et al. (2024)
Lactose (%)	4.5–5.5	4.5–5.0	4.5–5.0	E. S. I. El‐Agamy (2006), Seyiti et al. (2024)
Dry matter (g/L)	12–13	14–15	13–14	Benmeziane‐Derradji (2021), Seyiti et al. (2024)
Milk density (g/L)	1028–1032	1030–1033	1029–1032	E. S. I. El‐Agamy (2006), Muthukumaran et al. (2023)
pH	6.4–6.6	6.5–6.7	6.45–6.65	E. S. I. El‐Agamy (2006), Konuspayeva and Faye (2021)
Iodine Index	16.7	15.0	15.5–16.0	E. S. I. El‐Agamy (2006), Konuspayeva and Faye (2021)
Vitamin C (mg/L)	150–200	177–250	160–220	E. S. I. El‐Agamy (2006), Benmeziane‐Derradji (2021)
Vitamin A (μg/mL)	7.0–8.0	7.5–9.0	7.2–8.5	E. S. I. El‐Agamy (2006), Muthukumaran et al. (2023)
Carotene (μg/mL)	8–9	9–10	8.5–9.5	E. S. I. El‐Agamy (2006), Muthukumaran et al. (2023)
Calcium (g/L)	1.0–1.2	1.2–1.3	1.1–1.25	E. S. I. El‐Agamy (2006), Benmeziane‐Derradji (2021)
Phosphorus (g/L)	0.9–1.0	1.0–1.1	0.95–1.05	E. S. I. El‐Agamy (2006), Benmeziane‐Derradji (2021)
Milk yield per day	5–20 L	0.5–5 L	2–15 L	Khan and Iqbal (2001), Seyiti et al. (2024)
Total lactation yield	500–2000 L	500–1254 L	600–1800 L	Khan and Iqbal (2001), Seyiti et al. (2024)
Peak yield month	3rd–4th month	3rd–4th month	3rd–4th month (intermediate)	Ayadi et al. (2018), Seyiti et al. (2024)
Lactation period	10–14 months	14–18 months	12–16 months	Khan and Iqbal (2001), Seyiti et al. (2024)
Fatty acid composition	Higher C10, C18, C18:1 n‐7	Higher C14, C16, C18:1 n‐9	Intermediate C10–C18 fatty acids	Ayadi et al. (2018), Seyiti et al. (2024)
Seasonal variation	Unsaturated fatty acids decrease in winter; vaccenic acid ↑ in spring	Fat and dry matter slightly ↑ in winter; palmitic and stearic acids vary seasonally	Mixed seasonal variation; depends on dominant species	Ayadi et al. (2018), Seyiti et al. (2024)
Environmental adaptation	Hot, arid, drought‐resistant	Cold, arid, or steppe, cold‐resistant	Adapted to mixed climates; a combination of both species' traits	Sala (2022), Bornstein (2021)
Bioactive components	Lactoferrin, insulin‐like proteins, and antimicrobial peptides	Higher lactoferrin, vitamin C, and bioactive lipids	Intermediate levels may combine both species' bioactive	Badawy et al. (2025), Faustini et al. (2025)
Safety concerns	Zoonotic bacteria (Salmonella, *E. coli* ), prions risk low	Same zoonotic risks; slightly higher microbial load due to traditional milking	Intermediate risk depending on hygiene and processing	Swelum et al. (2021), Konuspayeva and Faye (2021)
Other uses	Meat, racing, transport, tourism	Meat, wool, transport, tourism	Meat, milk, transport	Faye (2015)
Nutritional/therapeutic potential	Supports immunity, metabolic health, moderate fat and protein	High vitamin C, fat, is good for malnutrition in harsh climates	Intermediate benefits combine the advantages of both species	Ali et al. (2019), Badawy et al. (2025)

### 3.1 Dromedary Camel ( *Camelus dromedarius* )

The dromedary camel, also known as the Arabian camel (Sala 2022), is well adapted to hot, arid desert environments, predominantly found in the Middle East, North Africa, and parts of South Asia. Adult dromedaries typically weigh between 400 and 600 kg and reach 1.8–2.0 m at the shoulder (Köhler‐Rollefson 1991). Their milk is relatively lighter, with a fat content of 3%–5%, protein 3%–4%, and lactose 4.5%–5.5% (E. S. I. El‐Agamy 2006). It is rich in vitamin C (150–200 mg/L) and calcium (1.0–1.2 g/L), with minerals like phosphorus at approximately 0.9–1.0 g/L (Benmeziane‐Derradji 2021). The fatty acid profile shows higher medium‐chain fatty acids, including capric (C10), stearic (C18), and oleic acid (C18:1 n‐7), with seasonal variations in unsaturated fatty acid content (Ayadi et al. 2018). Dromedary camels have a lactation period of 10–14 months, producing an average of 5–20 L of milk per day, with a total lactation yield ranging from 500 to 2000 L (Khan and Iqbal 2001). The milk is widely used for nutritional purposes, and dromedaries are also important for meat, transport, and racing in arid regions (Sala 2022).

Dromedary milk composition varies with breed, diet, and environment. Bioactive components such as lactoferrin, insulin‐like proteins, and exosomes differ across regions, influencing antimicrobial and immunomodulatory efficacy (Badawy et al. 2025). Processing methods like boiling, fermentation, and spray drying can significantly impact these bioactive components, for example, boiling may denature insulin‐like proteins, while fermentation may alter antimicrobial peptide activity, affecting therapeutic potential (Swelum et al. 2021).

### 3.2 Bactrian Camel ( *Camelus bactrianus* )

The Bactrian camel is a robust species adapted to cold desert and steppe environments in Central Asia, including Mongolia, Kazakhstan, China, and Russia (Bornstein 2021). Adult Bactrians generally weigh 450–650 kg and stand 1.7–2.2 m at the shoulder (Sala 2022). Their milk is richer than dromedary milk, with fat content ranging from 5.5%–6.7%, protein 3.5%–4.5%, and lactose 4.5%–5.0% (Seyiti et al. 2024). It contains higher levels of vitamin C (177–250 mg/L), calcium (1.2–1.3 g/L), and phosphorus (1.0–1.1 g/L) (E. S. I. El‐Agamy 2006). The fatty acid profile is dominated by long‐chain fatty acids, including myristic (C14), palmitic (C16), and oleic acid (C18:1 n‐9) and is affected by seasonal feed variations (Chen et al. 2025). Bactrian camels have a longer lactation period of 14–18 months but a lower daily milk yield, typically 0.5–5 L per day, with a total lactation yield of 500–1254 L (Seyiti et al. 2024). Bactrian milk is highly valued for its nutritional and therapeutic benefits, especially in harsh and cold climates, and these camels are also used for meat, wool, and transport purposes (Ali et al. 2019).

Bactrian milk exhibits higher bioactive substance content than dromedary milk, such as lactoferrin, exosomes, and insulin‐like proteins, enhancing its therapeutic potential (Faustini et al. 2025). Seasonal and regional feed differences strongly influence milk composition, including fat, protein, and vitamin content (Badawy et al. 2025). Processing methods similarly affect bioactive components, with heat treatment or prolonged fermentation potentially reducing the functional efficacy of these molecules (Muthukumaran et al. 2023).

### 3.3 Hybrid Camels (Bactrian × Dromedary)

Hybrid camels, produced by crossing Bactrian ( *Camelus bactrianus* ) and dromedary ( *Camelus dromedarius* ) camels, inherit traits from both parents (Dioli 2020; Faustini et al. 2025). They show intermediate milk yield and composition, balancing the high‐fat Bactrian milk and higher‐volume dromedary milk. Hybrids are more resilient to diverse climates and retain therapeutic milk components such as vitamins, insulin‐like proteins, and antimicrobial peptides (Sahoo 2020). Their adaptability and combined nutritional benefits make them valuable for dairy production and human consumption in regions where both species coexist (Sahoo 2020). Hybrid milk demonstrates variable levels of bioactive substances depending on the parental breed and local environmental conditions (Abdelazez et al. 2024). Processing methods such as fermentation, pasteurization, and drying can alter these components (Konuspayeva and Faye 2021).

## 4 Holistic Benefits of Camel Milk

Camel milk has been consumed for centuries in arid and semi‐arid regions, where it serves as both a staple food and a traditional remedy for various ailments (Arain et al. 2023). It is rich in essential nutrients, bioactive proteins, and immunomodulatory compounds, which contribute to its therapeutic potential. Its low lactose content, hypoallergenic properties, and presence of insulin‐like proteins make camel milk a suitable alternative for individuals with lactose intolerance and diabetes (Sahoo 2020). Moreover, its antioxidant, antimicrobial, and anti‐inflammatory activities further highlight its medicinal value (Arain et al. 2023).

### 4.1 Nutritional Benefits of Camel Milk

**FIGURE 1:**
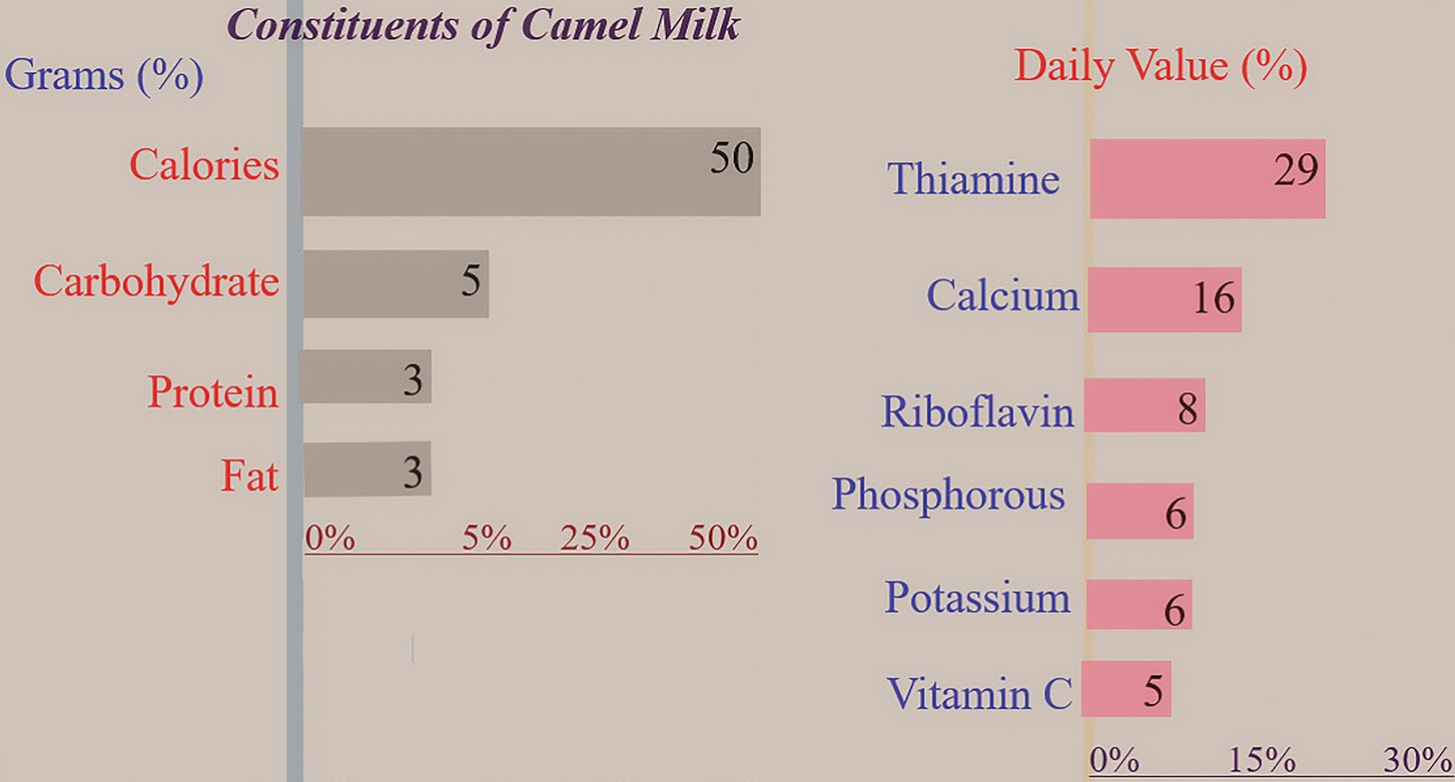
Macronutrient and selected micronutrient composition of camel milk, expressed in grams and percentage of recommended daily value.

Camel milk is a nutrient‐rich food recognized for its health and well‐being, and has attracted global attention due to its diverse nutritional advantages. Selected examples of these nutritional benefits are summarized below (Tables 2, 3, 4 and Figure 1). A survey by Cheikh Ismail et al. of 852 adults in the UAE examined patterns of camel milk consumption and perceptions. Approximately 60% of participants had tried camel milk, though only 25.1% were regular consumers. Yoghurt and flavored milk were commonly consumed, often with additives such as honey, turmeric, and sugar. Most respondents (57%) consumed less than one cup daily. Nutritional value (66.4%) and perceived medicinal properties (39.3%) were the primary reasons for consumption. Notable, 58.4% reported consuming unpasteurized milk, citing freshness (87.2%), immune benefits (41.6%), and higher nutrients (39.2%) as motivations (Cheikh Ismail et al. 2022).

**TABLE 2:** Nutritional composition of camel milk per 120 mL (½ cup), including macro‐ and micronutrients.

Constituents of camel milk									
Grams (%)				Daily value (%)					
Calories	Protein	Fat	Carbohydrate	Thiamine	Riboflavin	Calcium	Potassium	Phosphorus	Vitamin C
50	3	3	5	29	8	16	6	6	5

**TABLE 3:** Evidence supporting the nutritional benefits of camel milk and its derivatives.

Health benefit	References	Study design	Intervention	Outcomes	Findings
Nutritional value	Cheikh Ismail et al. (2022)	Survey, 852 adults, UAE	Camel milk consumption patterns	60% tried CM; 25.1% regular; < 1 cup/day for 57%; additives: honey, turmeric, sugar; knowledge low	Camel milk is valued for its nutritional and medicinal properties, but overall awareness is low
Functional foods	Farag et al. (2020)	Review	Kefir from camel/mixed milks with microbial fermentation	Kefir contains bacteria/yeasts; flavor, texture, and health effects are influenced by additives; antimicrobial, anticancer, gut, and antidiabetic benefits	Camel milk kefir shows health‐promoting properties and potential functional food applications
Nutrient composition	Ho et al. (2022)	Review	Comparison of camel vs. bovine vs. human milk	High bioactive compounds; tailored processing needed to preserve nutrients	Camel milk is nutritionally superior and requires optimized processing for functional products
Functional beverage	Ansari et al. 2024	Review	Probiotic camel milk	Contains LAB, Bifidobacteria, postbiotics, SCFAs, vitamins; antidiabetic, hypoallergenic, anticancer	Probiotic camel milk is a promising functional beverage with multiple health benefits
Bioactive compounds	Arain et al. (2023)	Review	Camel milk composition analysis	High medium‐chain fatty acids, whey protein, vitamin C, peptides, LAB, lactoferrin, lysozyme, immunoglobulins; antimicrobial, antioxidant, antidiabetic, hepatoprotective, nephroprotective	Camel milk is a functional food supporting immunity, metabolic health, and disease prevention
Musculoskeletal health	Kim et al. (2023)	Review	Milk exosomes	Antioxidant, anti‐inflammatory; may enhance drug therapy for sarcopenia/osteoporosis	Milk exosomes from camel milk may support muscle and bone health
Metabolomic profiling	Wang et al. (2023)	UHPLC‐Q‐TOF MS, 13 mammalian milks	1992 metabolites in 17 classes	KEGG pathways include ABC transporters, purine/pyrimidine metabolism	Camel milk provides key nutrients similar to human milk, supporting health
Gut microbiota/infant nutrition	Li et al. (2020)	In vitro fermentation	Cow, goat, camel, mare, human milk, infant formula	Increased Bifidobacterium and Lactobacillus; mare milk is similar to human milk	Camel milk supports beneficial gut microbiota, with potential for infant nutrition

**TABLE 4:** Mechanisms of action of camel milk components in metabolic and physiological processes.

Application	How it works	Mechanism	Responsible enzyme	References
Calories (energy source)	Provides essential energy for metabolic functions	Metabolized into glucose and used by the body for energy	Amylase, lipase	Kula and Tegegne (2016), Kumar et al. (2016a, 2016b), Kumar, Verma, et al. (2016)
Protein (building blocks)	Supports tissue repair, muscle growth, and enzyme activity	Broken down into amino acids during digestion, which are used for protein synthesis	Pepsin, trypsin	E. S. I. El‐Agamy (2006), Benmeziane‐Derradji (2021)
Fat (essential fats)	Provides essential fatty acids that promote brain and heart health	Emulsified by bile salts, then digested into fatty acids and glycerol	Lipase	Bakry et al. (2021), Alhassani (2024)
Carbohydrates (lactose)	Provides a primary energy source for the body, especially for children	Lactose is broken down into glucose and galactose by lactase	Lactase	E. S. I. El‐Agamy (2006), Kumar et al. (2016a, 2016b), Kumar, Verma, et al. (2016)
Thiamine (vitamin B1)	Essential for carbohydrate metabolism and nerve function	Converted into its active form (thiamine pyrophosphate) to act as a coenzyme in energy metabolism	Thiamine pyrophosphokinase	Kumar et al. (2016a, 2016b), Kumar, Verma, et al. (2016)
Riboflavin (vitamin B2)	Supports cellular energy production and antioxidants	Acts as a coenzyme in redox reactions for energy production	Flavokinase	Kumar et al. (2016a, 2016b), Kumar, Verma, et al. (2016)
Calcium (mineral)	Crucial for bone health, muscle contraction, and nerve function.	Absorbed in the intestines and stored in bones and teeth	Alkaline phosphatase	Kula and Tegegne (2016), Singh (2017)
Potassium (mineral)	Regulates fluid balance, nerve signals, and muscle function	Maintains proper cell function and helps transmit electrical impulses	Na+/K+ ATPase	Kula and Tegegne (2016)
Phosphorus (mineral)	Important for energy storage and cellular function	Forms part of ATP and other essential molecules	Phosphatases	Mostafidi et al. (2016)
Vitamin C	An antioxidant that aids in tissue repair and immune function	Protects cells from oxidative damage and supports collagen production	Ascorbate peroxidase	Singh (2017), Sharma et al. (2025)
Immunoglobulins	Boosts the immune system by neutralizing pathogens	Bind to pathogens and facilitate their destruction by immune cells	Proteases (to break down pathogens)	Abdelazez et al. (2024), Behrouz et al. (2024)
Lactoferrin (antibacterial agent)	Has antimicrobial properties and supports the immune response	Binds to iron, preventing bacterial growth and enhancing immune function		Benmeziane‐Derradji (2021), Abdelazez et al. (2024)

Farag et al. reviewed kefir made from various milks via microbial fermentation. Kefir contains bacteria and yeasts that shape chemical, nutritional, and sensory traits. Mixed milks and additives, like inulin, improve flavor, texture, and health properties. Fermentation generates lactic acid, ethanol, CO_2_, acetoin, and acetaldehyde, and storage further affects quality. Kefir shows antimicrobial, anticancer, gut, and antidiabetic effects, influenced by probiotics, prebiotics, and additives (Farag et al. 2020). (Ho et al. 2022) reported that camel milk is nutritionally superior to bovine milk and close to human milk, with high bioactive compounds.

Ansari et al. highlighted probiotic camel milk as a functional beverage containing LAB, Bifidobacteria, postbiotics, enzymes, SCFAs, vitamins, peptides, and organic acids (Ansari et al. 2024). It offers antidiabetic, hypoallergenic, and anticancer benefits. Arain et al. emphasized camel milk's composition: higher medium‐chain fatty acids, whey protein, vitamin C, and bioactive compounds (peptides, LAB, lactoferrin, lysozyme, casein, immunoglobulins). It exhibits antimicrobial, anti‐inflammatory, antioxidant, antidiabetic, hepatoprotective, nephroprotective, anticancer, and immunomodulatory effects. LAB enhances innate and adaptive immunity, supporting camel milk as a functional food for humans and animals (Arain et al. 2023).

#### 4.1.1 Rich in Nutrients

Camel milk contains minerals such as calcium, iron, zinc, folate, and potassium, as well as vitamins B and C (Kula 2016). Camel milk is a rich source of long‐chain and unsaturated fatty acids, which promote brain and cardiovascular health (Alhassani 2024). Camel milk also provides antioxidants that help reduce oxidative stress and free‐radical damage (Sharma et al. 2025). Camel milk contains modest amounts of various oligosaccharides that protect young children from viral infections, promote the growth of Bifidobacterium, and support nervous system development (Mohammadabadi et al. 2023). The total protein content of camel milk ranges from 2.15% to 4.90%, and it forms a softer gel due to differences in coagulum hardness (E. S. I. El‐Agamy 2006).

Camel milk has a higher ascorbic acid concentration and is particularly rich in calcium and iron (Singh 2017). Camel milk contains higher levels of insulin, whey acidic protein, peptidoglycan recognition protein, β‐lactoglobulin, casein micelles, whey proteins, and omega‐7 fatty acids (Benmeziane‐Derradji 2021). The total protein content of camel milk generally ranges between 2.1% and 4.9% (E. S. I. El‐Agamy 2006). Camel milk fat content ranges from 1.2% to 5.4%, with an average of approximately 3.29% (Bakry et al. 2021). Camel milk contains between 2.40% and 5.80% lactose (E. S. I. El‐Agamy 2006). In dromedary camel milk, the total mineral content ranges from 0.60% to 0.90% (Mostafidi et al. 2016). Due to the halophytic forage consumed by camels, such as Atriplex and Acacia, camel milk is a rich source of chloride, which may contribute to its salty taste (Abou El Ezz et al. 2015).

Iron, zinc, and copper concentrations in camel milk have been reported as 1.37, 2.19, and 0.44 mg/dL, respectively (Abdelrahman et al. 2022). According to the 2009 United States Drug Act report, consumption of 250 mL of camel milk provides approximately 15.5% of the recommended daily intake of vitamin B12, 8.25% of riboflavin (B2), 5.25% of vitamin A, and 10.5% each of vitamin C, thiamine (B1), and pyridoxine (B6) (Kumar et al. 2016a, 2016b; Kumar, Verma, et al. 2016).

Kim et al. summarized milk exosomes and their role in musculoskeletal health. Exosomes from human milk were confirmed via density (1.10–1.18 g/mL) and markers (MHC I/II, CD63, CD81, CD86). They show antioxidant and anti‐inflammatory properties and may support muscle and bone health. When combined with drugs, exosomes could enhance therapy for sarcopenia and osteoporosis (Kim et al. 2023). Wang et al. conducted metabolomic profiling of 13 mammalian milks using UHPLC‐Q‐TOF MS, identifying 1992 metabolites in 17 classes. KEGG analysis revealed pathways including ABC transporters, purine and pyrimidine metabolism, phosphotransferase systems, and galactose metabolism. Pig and goat milk were closest to human milk, followed by camel and cow milk, indicating camel milk provides important nutrients for human health (Wang et al. 2023).

#### 4.1.2 Identical to a Human Mother's Milk

Colostrum and breast milk share the same qualities as camel milk (Padayachee et al. 2025). As a healthy alternative to newborn formula, many parents supplement it with camel milk (Padayachee et al. 2025). Camel milk is particularly distinctive and full of immunoglobulins, which are defense proteins that bolster the immune system's resistance to diseases (Abdelazez et al. 2024). Because they are smaller than human immunoglobulins, the immunoglobulins found in camel milk can more readily enter bodily tissues (Behrouz et al. 2024).

Li et al. evaluated cow, goat, camel, mare, human milk, and infant formula via 24‐h in vitro fermentation. Mare milk produced the lowest gas pressure, similar to human milk; pH was comparable across all milks. 16S rRNA sequencing showed that all milk types increased Bifidobacterium and Lactobacillus proportionally to lactose content. Mare milk uniquely increased Akkermansia (Li et al. 2020).

#### 4.1.3 Limitations of Included Studies on the Nutritional Benefits of Camel Milk

However, several limitations exist. (Farag et al. 2020) reviewed kefir production and effects but lacked primary experimental data, and variability in preparation methods limits generalizability. (Ho et al. 2022) focused on compositional analysis without controlled intervention studies to confirm health outcomes. (Ansari et al. 2024) and (Arain et al. 2023) mainly relied on in vitro and animal studies, leaving human applicability uncertain. (Kim et al. 2023) reported biochemical properties of milk exosomes, but functional outcomes in humans remain speculative. (Wang et al. 2023) conducted cross‐species metabolomic comparisons, yet differences between species and sample variability may limit direct translation to human nutrition. Finally, (Li et al. 2020) used in vitro fermentation models, which may not fully replicate in vivo infant gut responses.

### 4.2 Health Benefits of Camel Milk

**FIGURE 2:**
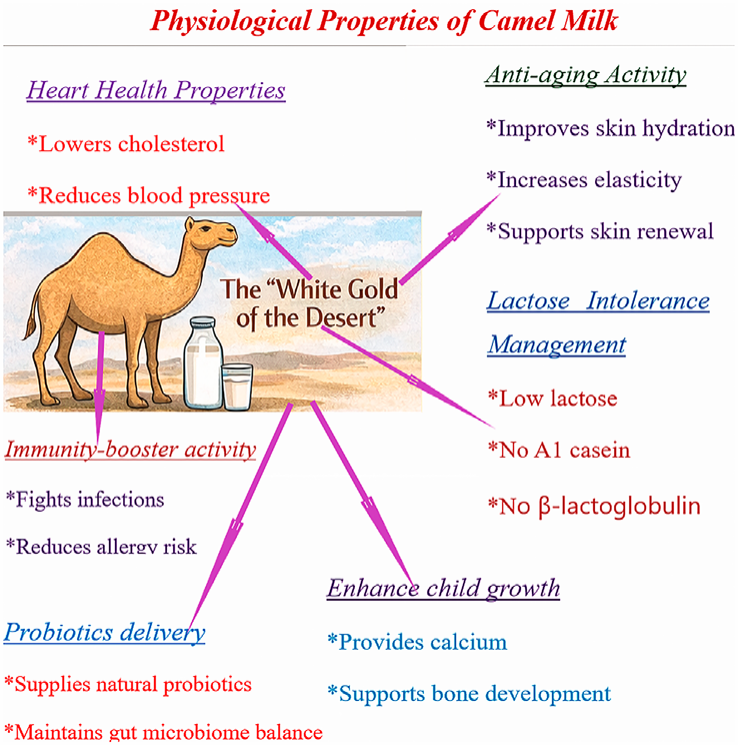
Proposed physiological and therapeutic mechanisms mediated by camel milk and its bioactive components.

Camel milk has been traditionally consumed for centuries due to its diverse nutritional properties (Muthukumaran et al. 2023). Numerous studies have reported its physiological benefits, which are summarized below (Tables 5 and 6; Figure 2) (Kumar, Verma, et al. 2016; Muthukumaran et al. 2023).

**TABLE 5:** Experimental and clinical evidence supporting the multifunctional physiological effects of camel milk and its derivatives.

Physiological effect	References	Sample and subjects	Intervention	Outcomes	Findings
CMPA activity	Navarrete‐Rodríguez et al. (2018)	15 children (1–18 years), cross‐over	Camel milk vs. amino‐acid formula	No adverse effects	Camel milk is a safe, tolerable, and palatable alternative for CMPA
Cardioprotective effects	Hamed, Chaari, et al. (2018)	28 mice, 4 groups	Fermented CM with *Lactococcus lactis* (100 mg/kg/day) ± CCl_4_	Reduced TBARS, protein carbonyls, cardiac markers, and ↑ antioxidants	Fermented camel milk protected against CCl_4_‐induced cardiac toxicity
	Hamed, Gargouri, et al. (2018)	Mice, acute CCl_4_‐induced cardiotoxicity	Camel milk	Reduced oxidative stress and cardiac biomarkers; preserved tissue	Camel milk prevented cardiac damage and oxidative stress
Skin protective effects	Jain et al. (2025)	In vitro (A431 cells) + mice skin carcinogenesis	Camel milk	Inhibited proliferation/migration; reduced tumor parameters; ↑ antioxidant activity	Camel milk exhibited antioxidant‐driven chemopreventive effects
Probiotic delivery	Devarajan et al. (2022)	Encapsulated probiotics in camel casein/gelatin	N/A	Enhanced survival in the GI tract; retained α‐glucosidase, α‐amylase, DPP‐IV, and pancreatic lipase inhibition	Camel milk matrices effectively deliver probiotics and preserve bioactivity
Wound healing effects	Badr et al. (2012)	STZ‐induced diabetic mice	Undenatured camel whey protein	Accelerated wound closure; restored cytokine/chemokine balance	Camel whey protein improved immune response and accelerated diabetic wound healing
Child growth activity	Muleta, Hailu, and Belachew (2021), Muleta, Hailu, Stoecker, et al. (2021)	388 children (24–59 months), cross‐sectional	Camel vs. bovine milk	Lower stunting/underweight in CM consumers	Camel milk is associated with better growth outcomes in children
Immuno‐protective effects	Aljutaily (2022)	Cyclophosphamide‐immunosuppressed rats	Turmeric‐camel milk (TCM)/Fermented TCM	↑ WBCs, IgG/A/M, antioxidant enzymes; ↓ MDA	Camel milk formulations enhanced immunity and oxidative stress protection
	Khalifa et al. (2023)	Chickens	Probiotic PM5 from camel milk	↓ Salmonella, ↑ butyric acid, improved mucosal immunity	Camel milk‐derived probiotics improved pathogen resistance and growth
	Badr et al. (2018)	Heat‐stressed mice (*n* = 45)	Camel whey protein	Restored ROS/cytokines; normalized T/B cells	Camel whey protein protected against heat‐stress‐induced immune disruption
Probiotics effects	Moussaid et al. (2023)	Moroccan raw camel milk	LAB	Tolerated GI conditions; inhibited pathogens; antioxidant activity	Selected LAB strains from camel milk are promising probiotics with antimicrobial and antioxidant effects
	Mokhtari et al. (2025)	144 LAB from Algerian camel milk	N/A	Tolerated gastric conditions; inhibited Listeria; antioxidant; GABA production	Camel milk LAB strains are safe, functional probiotics for food applications
	Sakr and Barakat (2025)	Freeze‐dried fermented CM + Ajwa date pulp	N/A	Low water activity, high dispersibility; α‐amylase/glucosidase inhibition; moderate Caco‐2 cytotoxicity	Camel milk‐date formulations enhance probiotic viability, antidiabetic potential, and functional food properties

**TABLE 6:** Major bioactive constituents of camel milk and their molecular and physiological mechanisms in human health.

Application	How it works	Key components in camel milk	Bioactive components responsible	References
Heart health	Camel milk improves heart health by lowering cholesterol and blood pressure	Camel milk's omega‐3 and orotic acids, along with lipase and protease enzymes, support heart health by aiding nutrient absorption	Orotic acid reduces cholesterol, while omega‐3 fatty acids have anti‐inflammatory effects that improve heart health	Almasri et al. (2024), Bakry et al. (2021), Aljumaah et al. (2025)
Rejuvenating (anti‐aging) properties	Camel milk promotes skin renewal, hydration, and elasticity, helping to reduce signs of aging	Camel milk's anti‐aging benefits are due to lactic acid, lanolin, collagen, elastin, and vitamin C, which help exfoliate, regenerate, firm, and protect the skin	Camel milk exfoliates the skin, firms it, retains moisture, and provides protective antioxidant effects	Oginga et al. (2024), Boubal et al. (2025), Saxena and Yadav (2020)
Lactose intolerance	Camel milk is more easily digestible due to its low lactose content and the absence of A1 casein and β‐lactoglobulin	Camel milk is low in lactose and free of A1 casein and β‐lactoglobulin, making it easy to digest	Low lactose and the absence of A1 casein/lactoglobulin make camel milk easy to digest and less allergenic	E. I. El‐Agamy (2009)
Height growth	Camel milk supports growth by providing calcium and other essential nutrients for bone and overall physical development	Camel milk promotes height growth through its calcium, essential amino acids, and alkaline phosphatase, which support bone development	Calcium and vitamins aid bone growth, and amino acids support protein synthesis and tissue development	Syman et al. (2024), Rafiq et al. (2015)
Natural immunity booster	Camel milk boosts immunity by helping fight infections and reducing allergy risk	Lactoferrin and immunoglobulins help boost immunity and fight infections	Lactoferrin blocks bacteria by binding iron; immunoglobulins fight pathogens	Niaz et al. (2019), Gul et al. (2015)
Natural probiotics	Camel milk supports gut health by supplying beneficial microbes that help maintain a balanced gut microbiome	Lactoferrin and camel milk proteins promote gut health by supporting beneficial bacteria	Lactoferrin in camel milk promotes gut health by supporting beneficial bacteria	Ali et al. (2024), Ansari et al. (2024)

#### 4.2.1 Enhance Heart Health

Omega‐3 fatty acids, which may play a role in lowering cholesterol and preventing the development of cardiovascular diseases, are abundant in camel milk (Almasri et al. 2024). Orotic acid, a component of camel milk, lowers cholesterol by reducing bad cholesterol in the body (Bakry et al. 2021; Aljumaah et al. 2025). It also aids in lowering high blood pressure and reducing the incidence of atherosclerosis, heart attacks, and strokes.

Hamed et al. evaluated the cardioprotective effects of fermented camel milk with *Lactococcus lactis* subsp. cremoris (FCM‐LLC) against carbon tetrachloride (CCl_4_)‐induced cardiac toxicity in mice. Twenty‐eight mice were divided into four groups: control, FCM‐LLC only (100 mg/kg/day for 15 days), CCl_4_ only (single 10 mL/kg i.p. dose on Day 14 in 0.3% olive oil), and FCM‐LLC + CCl_4_. CCl_4_ exposure increased lipid peroxidation (TBARS), protein carbonyl levels, and cardiac toxicity markers (ALT, LDH, CK, CK‐MB, and Troponin I), while reducing antioxidant enzymes (SOD, CAT, GPx), GSH, and vitamin C. Pretreatment with FCM‐LLC significantly attenuated oxidative stress, improved antioxidant defenses, and preserved heart tissue histology (Hamed, Chaari, et al. 2018).

Hamed et al. also demonstrated that camel milk administration protected mice from acute CCl_4_‐induced cardiotoxicity. Single‐dose CCl_4_ exposure elevated TBARS, protein carbonyls, and plasma markers (AST, LDH, CK, Troponin I), while lowering antioxidant levels in heart tissue. Camel milk treatment ameliorated both biochemical and histological alterations, indicating its efficacy in reducing oxidative stress and preventing cardiac tissue damage (Hamed, Gargouri, et al. 2018).

#### 4.2.2 Skin Protective Effects

Camel milk contains a higher concentration of alpha‐hydroxy acids, which aid in exfoliating dead skin cells, encourage their regeneration, and encourage the formation of new skin cells (Oginga et al. 2024; Boubal et al. 2025). These products include high concentrations of lactic acid, an alpha‐hydroxy acid subtype thought to be the most useful on sensitive skin. Lactic acid exfoliates dead skin cells and leaves the skin appearing clean and fresh (Saxena and Yadav 2020). This acid allows the skin to create ceramides and aids in moisture retention, giving the skin a plumper, redder, and healthier‐looking appearance. Camel milk also contains lanolin, collagen, and elastin, which help to firm, tighten, and elasticize skin as well as aid in moisture retention (Ijaz et al. 2025).

Camel milk has antiaging benefits because of its high vitamin C content, which protects collagen and has antioxidant and tissue‐repairing properties (Iqbal et al. 2024). Vitamin C improves the resilience and structural support of the skin, which aids in the healing of skin damage (Pullar et al. 2017). Antioxidant vitamin C decreases the rate of free‐radical damage, which results in wrinkles and dry skin (Wang et al. 2018). The liposome found in camel milk also has a cosmetic component that is employed to enhance the antiaging effect (Choi et al. 2014). Lanolin and other moisturizing ingredients in camel milk have a calming and soothing effect on the skin (Oginga et al. 2024). Elastin, vitamin C, and lanolin contribute to enhanced moisture, brightness, and soft skin. Camel milk has also become increasingly used in lotions, ointments, and masks (Bereda 2024).

Jain et al. investigated camel milk's chemopreventive potential against skin cancer using A431 cells and a two‐stage skin carcinogenesis model in mice. In vitro, camel milk exhibited dose‐dependent inhibition of cell proliferation, suppressed cell migration, and induced G1/S phase cell cycle arrest. In vivo, camel milk significantly reduced tumor parameters and histopathological lesions in skin and liver tissues. Biochemical analyses showed decreased lipid peroxidation and increased GSH, catalase, and SOD levels. Dose‐dependent enhancement of antioxidant activity (DPPH, ABTS, ferrous‐ion, and superoxide‐anion chelating) was observed, and GC–MS analysis identified numerous bioactive anticancer compounds (Jain et al. 2025). These findings support camel milk's antioxidant‐driven chemopreventive effects as a promising adjunct therapy.

Devarajan et al. encapsulated probiotic strains *Lactobacillus rhamnosus* MF00960, *Pediococcus pentosaceus* MF000967, and *Lactobacillus paracasei* DSM20258 in camel casein (CC), camel skin gelatin (CSG), CC: CSG (1:1), and sodium alginate. Encapsulated strains showed enhanced survival under simulated gastrointestinal digestion and higher thermal tolerance at 50°C and 70°C compared with free cells. Scanning electron microscopy revealed probiotics embedded in dense protein matrices, and FTIR analysis confirmed structural integrity. CC and CC: CSG matrices effectively preserved bioactivity, maintaining inhibitory effects against α‐glucosidase, α‐amylase, DPP‐IV, pancreatic lipase, and cholesteryl esterase (Devarajan et al. 2022). These findings demonstrate their suitability for probiotic encapsulation in food applications. Badr et al. studied undenatured camel whey protein (WP) on wound healing in streptozotocin‐induced type 1 diabetic mice. Diabetic mice exhibited delayed wound closure, reduced IL‐10, prolonged elevation of TNF‐α, IL‐1β, IL‐6, aberrant chemokine expression (MIP‐1α, MIP‐2, KC, CX3CL1), and altered growth factor TGF‐β levels. WP supplementation accelerated wound closure, restored cytokine balance, and normalized chemokine and TGF‐β expression, improving healing compared with untreated diabetic mice. WP also rescued the function of long‐lived, wound‐resident macrophages, enhancing phagocytosis, and activating STAT3, Akt, and NF‐κB pathways (Badr et al. 2012). These results demonstrate WP's capacity to improve immune response and accelerate diabetic wound healing.

#### 4.2.3 Enhance Child Growth

Camel milk contains significant amounts of calcium, vitamins, and minerals that are essential for overall body growth, including greater height (Gizachew et al. 2014). Methionine, valine, phenylalanine, arginine, and leucine are some of the amino acids that are present in camel milk in sufficient amounts to support healthy growth and development (Syman et al. 2024). For example, the amino acid phenylalanine “plays a key role in the biosynthesis of other amino acids and is significant in the structure and function of many proteins and enzymes” (Rafiq et al. 2015). One of the most fundamental parts of the body is protein, and camel milk is a good source of it (E. I. El‐Agamy 2009).

Muleta et al. reported that camel milk (CaM) consumption in pre‐school children was associated with a lower prevalence of stunting and underweight compared with children consuming bovine milk (BM). Regular CaM consumption was linked to better anthropometric outcomes, suggesting a protective effect of camel milk on growth in this population (Muleta, Hailu, and Belachew 2021; Muleta, Hailu, Stoecker, et al. 2021).

#### 4.2.4 Immunity‐Booster Activity

Compounds found in camel milk appear to combat a number of microbial‐based disorders (Elisha et al. 2025). The two primary components of camel milk are lactoferrin and immunoglobulins; it is possible that these two components are what give camel milk its immune‐strengthening properties (E. I. El‐Agamy 2009). Microorganisms associated with serious diseases, such as *Escherichia coli* , are prevented from multiplying by lactoferrin (Niaz et al. 2019). The disease‐fighting properties of immunoglobulins help to reduce allergy symptoms (Gul et al. 2015). Targeting foreign invaders and chemicals like antigens, antibody proteins work to eliminate them from the immune system (Gulbins and Lang 2001).

Aljutaily et al. evaluated turmeric‐camel milk (TCM) and fermented TCM (FTCM) in cyclophosphamide‐immunosuppressed rats. TCM or FTCM (10 or 20 mL/kg) for 2 weeks improved weight gain, feed efficiency, WBCs, lymphocytes, and neutrophils. IgG, IgA, and IgM levels increased significantly, while IL‐1β, IL‐6, IL‐10, IL‐13, and TNF‐α were enhanced dose‐dependently. FTCM was more potent than TCM. Both restored antioxidant enzymes (GSH, CAT, SOD) and reduced MDA, supporting immunity and oxidative stress protection (Aljutaily 2022). Khalifa et al. isolated probiotics from camel milk and tested them in chickens against Salmonella. PM5 ( *Bacillus subtilis* OQ913924) showed > 51% hydrophobicity and strong antimicrobial activity. Oral PM5 reduced Salmonella in the spleen, thymus, and feces (CFU 7.2 → 5.2) and increased butyric acid levels (Khalifa et al. 2023). It also decreased IL‐1β, CRP, and IFN‐γ, enhanced mucosal immunity, co‐aggregated with macrophages, and improved growth and resistance to pathogens.

Badr et al. studied CWP in 45 heat‐stressed mice. HS increased ROS and pro‐inflammatory cytokines (IL‐1β, IL‐6, and TNF‐α) and reduced IL‐2 and IL‐4. HS impaired AKT and IκB‐α phosphorylation, increased ATF‐3 and HSP70, and disrupted T/B cells. CWP restored ROS levels and cytokine balance, improved AKT/IκB‐α phosphorylation, and reduced ATF‐3, HSP70, and HSP90 (Badr et al. 2018). It also normalized T and B cell distribution, demonstrating immunoprotective effects via the PI3K‐AKT, NF‐κB, ATF‐3, and HSP70 pathways.

#### 4.2.5 Probiotics Delivery

Probiotics are beneficial microorganisms that reside in the gastrointestinal tract and promote gut health (Ali et al. 2024). Because it contains a variety of healthy bacteria, camel milk is a great source of probiotics (Ansari et al. 2024). Probiotics with physiological activity in the digestive system include lactoferrin and a camel milk protein that contains iron (Manaer et al. 2021).

Moussaid et al. identified LAB from Moroccan raw camel milk, including *Pediococcus pentosaceus* , *Enterococcus faecium* , and *Enterococcus durans* . All strains tolerated gastrointestinal conditions, including pH 2.5 (31.85%–96.52%), 0.3% bile (35.23%–99.05%), and pepsin (26.9%–90.96%). They also showed high auto‐aggregation (28.75%–95.9%) and hydrophobicity (80.47%–96.37%) and were able to coaggregate with pathogens. No hemolysis or antibiotic resistance was detected. LAB inhibited *S. aureus* , *P. aeruginosa* , *E. coli* , *B. subtilis* , and *S. enterica* , acidified milk (ΔpH 2.55/24 h), and improved antioxidant activity (36.77% DPPH). *P. pentosaceus* Pd24, Pd29, Pd38, *E. faecium* Ef18, and *E. durans* Ed22 were the most promising probiotics (Moussaid et al. 2023).

Mokhtari et al. evaluated 144 LAB from Algerian camel milk; 19 strains tolerated gastric conditions. Sequencing identified 15 Lactiplantibacillus plantarum, 3 *Lactobacillus gasseri* , and 1 *E. faecium* . All tolerated bile salts (94.86%–102.81%), lacked hemolytic, DNase, and gelatinase activity, and carried no virulence or antibiotic‐resistance genes. *L. gasseri* C1 showed high auto‐aggregation and hydrophobicity. Several strains, including *L. plantarum* A5, A8, BN2, and *L. gasseri* C1, inhibited *Listeria monocytogenes* . Most hydrolyzed bile salts; all had antioxidant activity, and 11 *L. plantarum* strains carried the gad gene for GABA production (Mokhtari et al. 2025).

Sakr et al. developed freeze‐dried fermented camel milk with Ajwa date pulp (12%–15%) and ABT‐5 starter culture, with or without *L. rhamnosus* B‐1937. Powders had low water activity (0.196–0.226) and high dispersibility (up to 72.73%). ADP improved rehydration, reduced clumping, and increased antioxidant content. Probiotics ( *L. acidophilus* and *B. bifidum* ) survived simulated gastrointestinal conditions. FCM15D + L showed antidiabetic activity (α‐amylase IC50 111.43 μg/mL, α‐glucosidase IC50 77.21 μg/mL) and moderate cytotoxicity against Caco‐2 cells (IC50 82.22 μg/mL). ADP‐enriched FCM enhances probiotic viability, antidiabetic potential, and functional properties, suggesting camel milk‐date synergy as a promising functional food (Sakr and Barakat 2025).

#### 4.2.6 Limitations of Included Studies on the Health Benefits of Camel Milks

However, these studies have several limitations. Many studies were animal‐based (Hamed, Chaari, et al. 2018; Hamed, Gargouri, et al. 2018; Jain et al. 2025; Badr et al. 2012; Badr et al. 2018; Aljutaily 2022), which limits direct translation to humans. Small sample sizes reduce statistical power (Hamed, Chaari, et al. 2018; Hamed, Gargouri, et al. 2018). Several studies lack randomization, blinding, or control for confounders, increasing the risk of bias. Short intervention durations and absence of long‐term follow‐up make it difficult to assess sustained effects or safety. Observational human studies (Muleta, Hailu, and Belachew 2021; Muleta, Hailu, Stoecker, et al. 2021) cannot establish causality, and potential confounders, such as diet and socioeconomic status, were not fully controlled. In studies on probiotics and functional foods (Devarajan et al. 2022; Moussaid et al. 2023; Mokhtari et al. 2025; Sakr and Barakat 2025), findings are mostly in vitro or in animal models, limiting human applicability.

### 4.3 Therapeutic Benefits of Camel Milk

**FIGURE 3:**
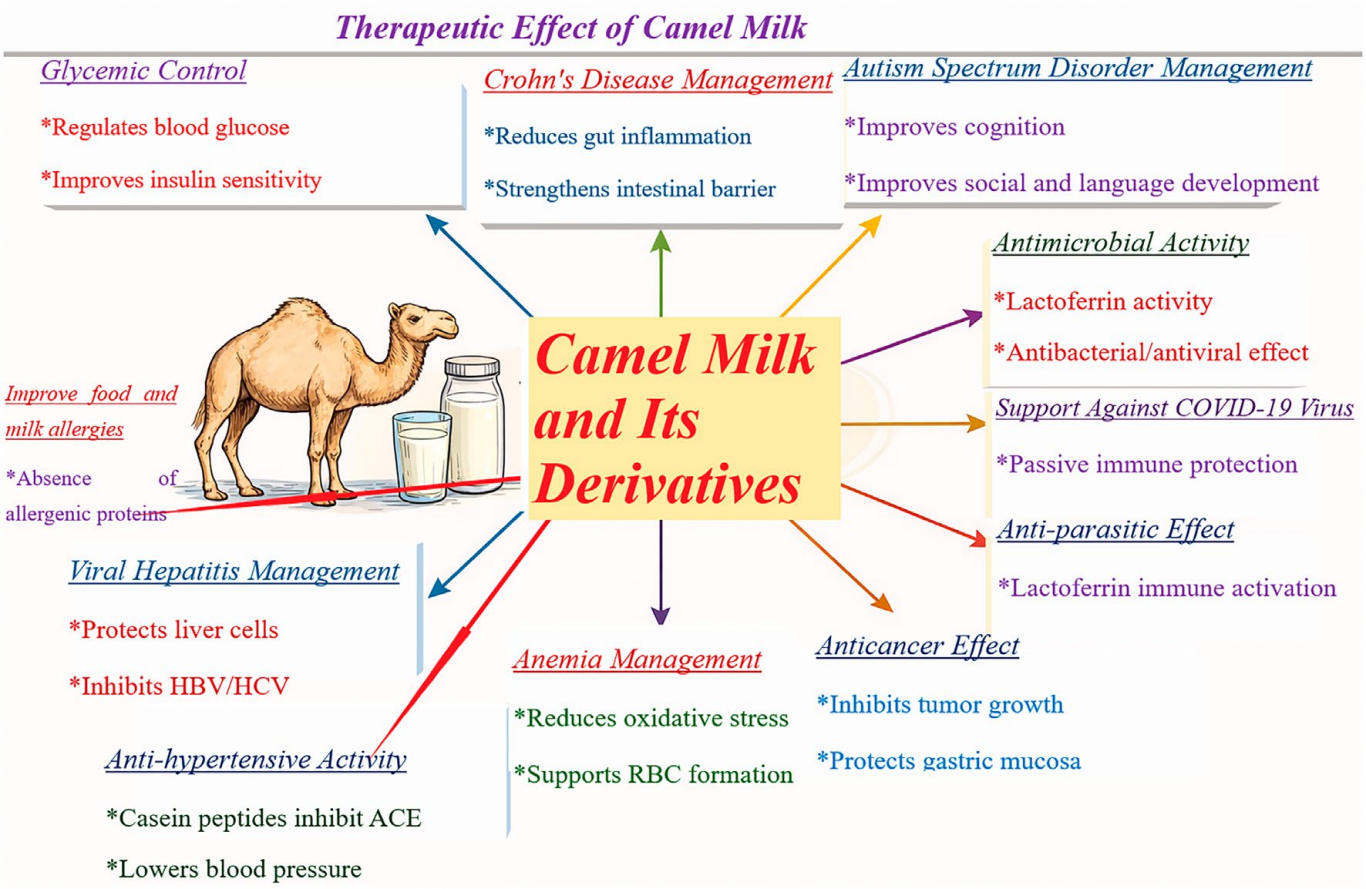
Multifunctional therapeutic actions of camel milk supported by experimental and clinical evidence.

In traditional medicine, local healers have reported that a mixture of camel milk and urine possesses potential anticancer properties (Alebie et al. 2017). Camel milk itself exhibits a wide range of therapeutic benefits, including antibacterial, anticarcinogenic, antioxidant, antihypertensive, and antidiabetic properties (Muthukumaran et al. 2023). The findings are summarized as follows: (Tables 7 and 8; Figure 3).

**TABLE 7:** Synthesis of experimental, clinical, and translational evidence on the therapeutic effects of camel milk and its derivatives.

Therapeutic effect	References	Study design/sample	Intervention	Duration	Key outcomes	Final findings
Anti‐diabetic effects	Wang et al. (2009)	32 obese diabetic rats +12 T2DM patients	Camel milk (raw, 500 mL/day) ± rosiglitazone	14 weeks (rats), 14 weeks (humans)	Reduced glucose, triglycerides, cholesterol, and plasma insulin; increased body weight in humans	Camel milk improved glycemic control and lipid profile; enhanced rosiglitazone effects
	Sboui et al. (2022)	60 T2DM patients	500 mL/day raw camel milk + OAD	3 months	Reduced FBG, PPG, HbA1c (~30%), cholesterol, triglycerides; urea/creatinine unchanged	Camel milk significantly improved glycemic and lipid parameters in T2DM patients
	Zheng et al. (2021)	27 patients, RCT	Camel milk powder 10 g BID vs. cow milk powder	4 weeks	Reduced fasting/postprandial glucose, total cholesterol; decreased resistin/lipocalin‐2; increased osteocrin, amylin, GLP‐1; beneficial gut microbiota modulation	Camel milk powder lowered glucose, improved lipid profile, and modulated gut microbiota
Autism spectrum disorder management	Al‐Ayadhi et al. (2022)	64 children (2–12 years), blinded	Raw/boiled CM vs. cow milk	2 weeks	Reduced TNF‐α; improved SRS scores and GI symptoms	Camel milk improved inflammation, behavior, and gastrointestinal symptoms
	Bashir and Al‐Ayadhi (2014)	45 children, DB‐RCT	Raw/boiled CM vs. cow milk	2 weeks	Decreased serum TARC; CARS improvement only in raw CM	Raw camel milk improved behavioral scores and reduced inflammation
	Kandeel et al. (2024)	5 RCTs, 299 children	CM consumption	Variable	Non‐significant pooled CARS improvement; qualitative improvements in social behavior, anti‐inflammatory/antioxidant biomarkers	Camel milk improved social behaviors and biomarker profiles despite non‐significant CARS scores
Crohn's disease management	Rosenheck et al. (2012)	Case report, 22 years male	Raw CM 8 oz. TID	1 week–1 year	Resolution of diarrhea, improved colonoscopy findings, and weight gain	Camel milk improved Crohn's disease symptoms and promoted tissue healing
	Zhao and Xiao (2025)	MR study	Dairy products, including milk	N/A	Whole milk reduced CD risk; mediated by isoleucine/valine	Milk intake is associated with reduced Crohn's disease risk via amino acid mediation
Antimicrobial Effects	Shaban et al. (2023)	In vitro	Camel milk exosomes	N/A	Bacteriostatic/fungistatic; induced apoptosis in HepG2/CaCO_2_; increased ROS, decreased Nrf2/HO‐1	CM exosomes selectively inhibited microbes and cancer cells without harming normal cells
	Hussein and Muhialdin (2021)	Fermented CM with *L. plantarum*	RP‐HPLC, LC–MS/MS	24 h	21 fractions; 30 peptides; strongest activity in fraction 14 against *E. coli* / *S. aureus*	Fermented camel milk generated potent antimicrobial peptides
	Abbes et al. (2021)	CM casein hydrolysates in vitro	Pepsin hydrolysates	5–180 min	Strong antioxidant activity; inhibited Coxsackie B6 virus in A549/Vero	Camel milk hydrolysates had antioxidant and antiviral effects
Supportive against COVID‐19	Masika et al. (2025)	Camel colostrum + RCT	22 serum/12 colostrum; 43 COVID‐19 patients	N/A	Neutralized MERS‐CoV in vitro; limited effect on SARS‐CoV‐2 viral load	Camel colostrum neutralized MERS‐CoV but limited SARS‐CoV‐2 efficacy in vivo
Anti‐cancer Effects	Kousar et al. (2024)	In vitro, TNBC cells	DTX‐loaded CM fat globule liposomes	N/A	Dose‐dependent cytotoxicity; 60.2% inhibition at 180 μg/mL; stable for 24 h	Camel milk liposomes enhanced docetaxel delivery and anticancer effect
	Krishnankutty et al. (2018)	In vitro, HCT116, MCF‐7	Camel milk	N/A	Reduced proliferation, migration; induced autophagy (LC3‐II, p62, Atg 5–12)	Camel milk inhibited cancer cell growth and induced autophagy
	Hasson et al. (2015)	In vitro, BT‐474, HEp‐2	Lyophilized CM	24–72 h	Suppressed proliferation; induced intrinsic/extrinsic apoptosis; ROS, HO‐1 ↑	Camel milk suppressed cancer cell growth via apoptosis pathways
Respiratory Protective Effects	Behrouz et al. (2024)	35 male Wistar rats, CS‐induced COPD	CM 4–8 mL/kg	N/A	Decreased WBC, TNF‐α, MDA; increased CAT, SOD, thiols; high‐dose CM > dexamethasone	Camel milk reduced inflammation and oxidative stress in COPD
	Bakhtiari et al. (2021)	60 children > 6 y, DB‐RCT	200 mL CM/day	2 months	Reduced inhaled corticosteroid, SABA, LABA use; FEV1/FEV1‐FVC not significant	Camel milk improved asthma management and reduced medication reliance
	Ravaghi et al. (2019)	46 patients, RC	250 mL CM BID + standard therapy	3 months	Improved FEV1, FEV1%, CAT scores	Camel milk improved lung function and symptom control
Anemia management	Abdurahman and Gashu (2021)	332 children, 6–59 months	Camel vs. cow milk	Cross‐sectional	Higher hemoglobin in CM (9.6 ± 1.8 vs. 9.1 ± 2.2 g/dL)	Camel milk improved hemoglobin levels in children
	Muleta, Hailu, and Belachew 2021; Muleta, Hailu, Stoecker, et al. 2021	388 children, 24–59 months	Camel vs. bovine milk	Cross‐sectional	Lower anemia prevalence in CM (42.7% vs. 75.4%); BM AOR 3.12	Camel milk consumption reduced anemia prevalence
Viral hepatitis management	Redwan et al. (2017)	17 HCV patients	250 mL/day CM	4 months	ALT ↓ in 88%, AST ↓ all, viral load ↓ 76.47%, IgG1 ↓	Camel milk improved liver enzymes, immunity, and viral clearance in most patients
Anti‐parasitic Effects	Maghraby et al. (2005)	*S. mansoni* ‐infected mice	Colostral and mature CM	6 weeks	Protection rates 12.81% (colostral) and 31.60% (mature); GST ↑; IgG ↑	Camel milk provided partial protection and immunomodulation against schistosomiasis
Antihypertensive Effects	Alshuniaber et al. (2021)	36 Wistar rats, 10% fructose	CM hydrolysate 800–1200 mg/kg	21 days	Reduced systolic/diastolic BP; ACE ↓; glucose, insulin, TG, cholesterol normalized	CM hydrolysate lowered BP and improved metabolic parameters
	Alshuniaber et al. (2022)	48 high‐fructose rats	CM protein hydrolysate vs. intact CM	21 weeks	Reduced glucose, insulin, cholesterol, TG, ALT, AST, BP; CMH > ICM	Camel milk hydrolysate was more effective than intact milk in metabolic and cardiovascular improvements

**TABLE 8:** Molecular and physiological pathways mediating the multifunctional therapeutic effects of camel milk.

Health conditions	How it acts	Key components in camel milk	Bioactive responsible	Enzymes	References
Diabetes mellitus	Camel milk exhibits antidiabetic effects by helping regulate blood glucose levels, improving insulin sensitivity, and reducing the need for exogenous insulin therapy.	Insulin‐like protein, casein, zinc, lactoferrin.	Camel milk supports glucose control by enhancing insulin action and absorption, thereby helping to maintain normal blood sugar levels.	Lactoferrin helps regulate insulin activity, while casein and insulin‐like proteins play important roles in controlling blood glucose levels.	(Bereda 2021), Anwar et al. (2022), Gastaldelli (2011), Ejtahed et al. (2015), Roy et al. (2020)
Autism spectrum disorder	Camel milk may improve cognitive function, motor skills, and social and language development in children with ASD.	Low beta‐casein, absence of beta‐lactoglobulin, protective proteins.	Camel milk supports brain development and immune function in autism due to its low beta‐casein content and absence of beta‐lactoglobulin.	Immunoglobulins in camel milk may help modulate the immune system and reduce ASD symptoms.	van Sadelhoff et al. (2019), Fiedorowicz et al. (2022), Mukani (2024)
Crohn's disease	Camel milk helps reduce gut inflammation and strengthens the intestinal barrier.	Anti‐inflammatory proteins, occludin, and claudin.	Camel milk helps regulate gut inflammation and supports the repair of the intestinal barrier in Crohn's disease.	Lactoferrin and casein proteins contribute to reducing inflammation.	Behrouz et al. (2022), Al‐Omari et al. (2019)
Anti‐bacterial and anti‐viral properties	Camel milk has strong antibacterial and antiviral properties due to lactoferrin and other antimicrobial enzymes.	Lactoferrin, lactoperoxidase, peptidoglycan recognition protein (PGRP), and immunoglobulins.	Camel milk combats bacteria and viruses through lactoferrin, lactoperoxidase, and immunoglobulin.	Lactoperoxidase and PGRP fight bacteria and support immunity.	Arain et al. (2023), Silva et al. (2020), Almehdar et al. (2023), Allam et al. (2025)
Support against COVID‐19	Camel milk provides passive immunity against COVID‐19.	Lactoferrin neutralizing antibodies.	Lactoferrin blocks the entry of SARS‐CoV‐2 into cells	Lactoferrin helps protect against viral infections, including COVID‐19	Chouchane et al. (2021), Mohammadabadi (2021)
Cancer, tumors, and ulcers	Camel milk helps prevent cancer growth and protects the stomach lining.	Lactoperoxidase, vitamins (C, A, B‐2, E), magnesium, zinc.	Lactoperoxidase and nutrients in camel milk help prevent tumor growth and protect the gut.	Lactoperoxidase is a key enzyme involved in inhibiting tumor formation.	Özhan et al. (2025), Abdel‐Mobdy et al. (2021), Arain et al. (2023)
Anti‐inflammatory effect	Camel milk helps reduce inflammation and oxidative stress.	Omega‐3 fatty acids, vitamins (A, B‐2, C), magnesium, zinc.	Omega‐3 fatty acids, vitamins A and C, and magnesium work together to reduce inflammation and oxidative stress.	Lactoferrin reduces inflammation, while magnesium enhances antioxidant defenses.	Alagawany et al. (2022), Vincenzetti et al. (2022), Singh et al. (2023)
Iron deficiency anemia	Camel milk prevents iron deficiency by supplying iron for red blood cells.	Iron, lactoferrin.	Lactoferrin is an iron‐binding protein that improves iron absorption and helps prevent iron deficiency anemia.	Lactoferrin regulates and transports iron, making it bioavailable.	Kuhn et al. (2017), Chikha and Faye (2025), Niaz et al. (2019)
Hepatitis	Camel milk protects liver function and inhibits hepatitis B and C viruses.	Ascorbic acid, lactoferrin.	Lactoferrin in camel milk inhibits hepatitis virus replication in liver cells, contributing to its therapeutic effect.	Lactoferrin enhances the immune‐modulating and antiviral effects of camel milk.	Shakeel et al. (2022), Khan et al. (2021)
Anti‐schistosomal effects	Camel milk lactoferrin protects against *Schistosoma mansoni* and enhances immune function.	Lactoferrin, colostrum proteins.	Lactoferrin and colostrum aid immune defense against *Schistosoma mansoni* .	Lactoferrin supports immunity and helps detoxify the body.	Reyes‐López et al. (2022), Almasri et al. (2024)
ACE inhibition (blood pressure regulation)	Casein in camel milk helps lower blood pressure by inhibiting ACE.	Casein, β‐casein.	Casein and β‐casein in camel milk inhibit ACE, helping to regulate blood pressure.	Enzymes release ACE‐inhibitory peptides from camel milk proteins.	Bakhle (2020), Berecek et al. (2018), Alhaj et al. (2024)
Food and milk allergies	Camel milk has a lower risk of causing allergies in children due to its unique protein composition.	Immunoglobulins, absence of β‐lactoglobulin.	Camel milk proteins help regulate the immune system and reduce allergic reactions.	Camel milk immunoglobulins help control allergies.	Arain et al. (2023), Zuhri and Wilda (2024), Acharya et al. (2025)

#### 4.3.1 Complementary for Diabetes Mellitus Therapy

Diabetes mellitus is a chronic condition characterized by elevated blood sugar levels (Bereda 2021). It occurs when pancreatic beta cells fail to produce sufficient insulin or when the body cannot use insulin effectively. Chronic hyperglycemia and abnormalities in the metabolism of carbohydrates, fats, and proteins are hallmarks of Type 1 diabetes mellitus, an autoimmune condition that affects just one organ (Popoviciu et al. 2023). These symptoms are linked to an insulin shortage. Camel milk helps to improve fasting blood glucose, glycosylated hemoglobin, serum anti‐insulin antibodies, and body mass index while lowering the amount of insulin needed to induce glycemic control (Agrawal et al. 2011). Camel milk has sufficient amounts of insulin as well as an insulin‐like protein with a 52 microunit/ml concentration that is used to sustain and prevent diabetes mellitus (Bereda 2024). The casein concentration of camel milk is unusual and appears to include an insulin‐like protein that resists intestinal digestion and absorbs more quickly into the blood (Anwar et al. 2022). Peripheral insulin resistance and insufficient insulin production by pancreatic beta cells are two characteristics of Type 2 diabetes mellitus (Roy et al. 2020). Camel milk can aid in reducing insulin resistance and blood sugar levels (Kahn 2003). Zinc minerals are abundant in camel milk, which may assist in increasing insulin sensitivity (Gastaldelli 2011). When people with Type 2 diabetes mellitus consume camel milk on a regular basis, their blood sugar levels are adjusted due to changes in their lipid profile, blood pressure, and insulin levels (Ejtahed et al. 2015). Additionally, casein, present in milk as large micellar aggregates, helps protect pepsin and insulin molecules from degradation in the stomach, thereby preserving their biological activity (Roy et al. 2020).

Wang et al. evaluated the camel milk as an adjunct to routine Type 2 diabetes management in 32 obese diabetic rats and 12 patients. In animal models, 14 weeks of camel milk supplementation significantly improved blood glucose, triglycerides, total cholesterol, and plasma insulin compared with untreated diabetic controls. The combination of rosiglitazone and camel milk produced greater reductions in triglycerides and plasma insulin than rosiglitazone alone. In patients, daily consumption of 500 mL of raw camel milk led to increased body weight and significant reductions in mean blood glucose, plasma insulin, antidiabetic drug dose, triglycerides, and total cholesterol (Wang et al. 2009). Sboui et al. investigated 60 patients with type 2 diabetes receiving oral antidiabetic agents, of whom one group additionally consumed 500 mL/day of raw camel milk for 3 months. Camel milk intake significantly reduced fasting blood glucose (from 9.89 ± 0.98 to 6.13 ± 0.55 mmol/L) and postprandial glucose (from 15.89 ± 4.34 to 7.44 ± 1.02 mmol/L). HbA1c decreased from 9.44% ± 0.16% to 6.61% ± 0.14%, representing a 30% reduction. Total cholesterol and triglycerides also decreased significantly, while urea and creatinine levels remained unchanged (Sboui et al. 2022).

Zheng et al. conducted a randomized, double‐blind, placebo‐controlled trial in patients with type 2 diabetes who received camel milk powder (10 g twice daily for 4 weeks). Camel milk powder significantly reduced fasting and 2‐h postprandial blood glucose and total cholesterol, decreased resistin and lipocalin‐2, increased osteocrin, amylin, and GLP‐1 levels and enhanced the relative abundance of Clostridium sensu stricto 1 and [Eubacterium] eligens group, indicating beneficial modulation of gut microbiota (Zheng et al. 2021).

#### 4.3.2 Camel Milk in Autism Spectrum Disorder Management

The term “autism spectrum disorder” is used to describe a number of neurodevelopmental problems that can affect social interaction and repetitive or aberrant behaviors (Lombardi and Troisi 2021). Numerous autoimmune diseases, which affect an intestinal enzyme involved in the production of amino acids, are the primary cause of many autistic disorders (van Sadelhoff et al. 2019). Casomorphine, a potent opioid, can occasionally form from the original beta‐casein and beta‐lactoglobulin in casein (Fiedorowicz et al. 2022). Camel milk is good for maintaining the immune system and promoting brain development due to its lower beta‐casein level, absence of beta‐lactoglobulin, and presence of protective proteins (Mukani 2024). Regular consumption of camel milk by individuals with autism may support motor skill development, improve behavioral and cognitive abilities, and reduce oxidative stress (Kula 2016). It may also enhance social interaction, communication, and language skills.

Al‐Ayadhi et al. conducted a blinded study in 64 children with ASD (2–12 years) who received raw camel milk, boiled camel milk, or cow milk as a placebo for 2 weeks. Both raw and boiled camel milk significantly reduced plasma TNF‐α levels, improved SRS scores, and alleviated gastrointestinal symptoms, whereas the placebo group showed no improvements (Al‐Ayadhi et al. 2022). Bashir et al. conducted a double‐blind randomized trial in 45 children with ASD who consumed 500 mL/day of boiled camel milk, raw camel milk, or cow milk placebo for 2 weeks. Both the boiled camel milk and raw camel milk groups reduced serum TARC levels, and a significant improvement in CARS scores was observed only in the raw camel milk group (Bashir and Al‐Ayadhi 2014).

Kandeel et al. conducted a meta‐analysis of five randomized controlled trials including 299 children with ASD. Camel milk consumption resulted in a reduction in mean CARS scores compared with controls, with raw camel milk demonstrating a greater effect than boiled camel milk. Qualitative synthesis also indicated improvements in social behaviors, along with increases in anti‐inflammatory, antioxidant, and immunomodulatory biomarkers (Kandeel et al. 2024).

#### 4.3.3 Crohn's Disease Management

Crohn's disease is a chronic inflammatory disorder of the gastrointestinal tract or gut, characterized by weight loss, abdominal pain, diarrhea, and systemic manifestations such as fatigue, skin lesions, arthritis, and eye inflammation (Włodarczyk et al. 2023). Weight loss, stomach discomfort, vomiting, or diarrhea are just a few of the signs and symptoms of Crohn's disease (Feuerstein et al. 2017). It can also lead to other health issues like eye inflammation, fatigue, poor focus, skin rashes, and arthritis. By limiting the overexpression of pro‐inflammatory cytokines in the colon, camel milk can reduce the inflammatory response (Behrouz et al. 2022). In order to re‐establish the function of the intestinal barrier, camel milk also increased the expression of occludin and claudin (Al‐Omari et al. 2019).

Rosenheck et al. reported a case of a 22‐year‐old male with severe Crohn's disease (CDAI 400) who declined biologic therapy and instead consumed 8 oz. of camel milk three times daily, discontinuing budesonide after ~1 week. The patient experienced complete resolution of symptoms, except for transient hip arthritis. A 1‐year follow‐up colonoscopy showed marked improvement in ileal ulcers and colonic inflammation, along with an increase in body weight. The therapeutic effects were attributed to camel milk's ~2% fat (mainly omega fatty acids) and bioactive proteins with antimicrobial and tissue‐repair properties (Rosenheck et al. 2012).

Zhao et al. used Mendelian randomization to evaluate the effect of six dairy products on Crohn's disease risk. Whole milk intake was associated with a reduced risk of Crohn's disease, partially mediated by serum isoleucine and valine, which contributed to 38.7% and 12.1% of the protective effect, respectively (Zhao and Xiao 2025).

#### 4.3.4 Antibacterial and Antiviral Effects of Camel Milk

Camel milk contains multiple bioactive components with antimicrobial and immune‐modulating properties (Arain et al. 2023). Lactoferrin, present at 95–250 mg/dL, inhibits microbial growth by binding iron, limiting its availability to pathogens such as *Salmonella typhimurium* (Mohammadabadi 2020; Arain et al. 2023). Lactoperoxidase exhibits bacteriostatic activity against Gram‐negative bacteria and *Lactococcus lactis* , while peptidoglycan recognition protein (PGRP) provides broad antimicrobial effects and stimulates immune responses (Silva et al. 2020; Almehdar et al. 2023, 2020). Camel milk has demonstrated therapeutic potential in tuberculosis, including multidrug‐resistant forms, and can serve as an adjuvant dietary supplement (Alkhulaifi et al. 2024). Camel milk also provides antirotavirus activity, particularly in children under five. Secretory immunoglobulin A (sIgA) and immunoglobulin G (IgG), especially in colostrum, inhibit rotaviruses from human and bovine sources, reducing diarrhea (Allam et al. 2025; Troeger et al. 2018; De Greve and Fioravanti 2024; Elbarbary et al. 2014).

Exosomes derived from camel milk (CM‐EXO) exhibit selective antibacterial, antifungal, and anticancer effects. At 6 mg/mL, CM‐EXO displayed bacteriostatic activity against Gram‐negative bacteria (*E. coli, P. aeruginosa, P. mirabilis*) and fungistatic activity against *Candida albicans* , while remaining non‐toxic to normal Vero cells. In cancer cell lines (HepG2, CaCo2), CM‐EXO induced apoptosis, increased Bax and caspase‐3, decreased Bcl‐2, elevated intracellular ROS, and downregulated NrF2 and HO‐1 (Shaban et al. 2023).

Fermented camel milk with *Lactobacillus plantarum* enhanced antibacterial activity by lowering pH and generating antimicrobial peptides, particularly fraction 14, which inhibited *E. coli* and *S. aureus* . LC–MS/MS identified 30 novel peptides contributing to this activity (Hussein and Muhialdin 2021). Additionally, camel milk casein hydrolysates demonstrated strong antioxidant and antiviral activity, effectively inhibiting Coxsackie virus B6 in A549 and Vero cells (Abbes et al. 2021).

Tahir et al. conducted a quasi‐experimental study to evaluate the effects of camel milk in tuberculosis patients. Twelve patients received 250 mL of camel milk twice daily for 40 days. Camel milk significantly increased BMI, reduced ESR more than other milk types, and led to sputum smear conversion to negative in five patients (Tahir et al. 2022). These findings suggest that camel milk may help reduce tuberculosis‐related disease activity.

#### 4.3.5 Supportive Against COVID‐19

Camel milk contains antiviral characteristics that can help the body fight sickness (Kula 2016). Due to the neutralizing antibodies in camel milk, it can provide passive immunity and be used to treat COVID‐19 infection (Chouchane et al. 2021; Jawhara 2020). It also provides prophylaxis for people who are at risk of contracting SARS‐CoV‐2. Lactoferrins have the ability to bind to heparan sulfate and prevent viral infections and pandemics (Mohammadabadi 2021). Camel milk has a high concentration of lactoferrin, which works to block SARS‐CoV‐2 entry and infection into host cells (Mohammadabadi 2021).

Masika et al. investigated camel colostrum‐derived antibodies for MERS‐CoV prophylaxis and SARS‐CoV‐2 control. Approximately 72% of camels in Israel are MERS‐CoV seropositive, suggesting therapeutic potential. Using ELISA, 22 serum and 12 colostrum samples showed MERS‐CoV spike antibodies with partial cross‐reactivity to SARS‐CoV‐2. Neutralization titers were ~1:500 for MERS‐CoV and ≤ 1:120 for SARS‐CoV‐2. In a randomized placebo‐controlled trial of 43 COVID‐19 patients, colostrum did not significantly reduce viral load or infectivity at 24 h. The study concludes that camel colostrum neutralizes MERS‐CoV in vitro, but more concentrated or viscous formulations should be explored for prophylaxis (Masika et al. 2025).

#### 4.3.6 Cancer, Tumors, and Ulcers Effects

By acting on thyroid peroxidase enzymes that are naturally present in camel milk, the lactoperoxidase enzyme inhibits the formation of tumors and is linked to the iodination of thyroid hormones (Özhan et al. 2025). The highest concentration of these enzymes also affects the reduction of breast cancer cell metastasis (Abdel‐Mobdy et al. 2021). Camel milk can treat cancer by binding to the tumors and eliminating the malignant cells without harming healthy tissue (Alebie et al. 2017). Due to its high concentrations of vitamins C, A, B‐2, and E (and its acidic pH), as well as the minerals magnesium and zinc, camel milk has been proven to possess antiulcer qualities (Arain et al. 2023). Camel milk considerably reduced indomethacin‐induced stomach ulcers, demonstrating an antiulcerogenic action associated with its gastroprotective function (Belhocine et al. 2017).

Kousar et al. encapsulated Docetaxel (DTX) into camel milk fat globule‐derived liposomes for delivery in triple‐negative breast cancer cells. In silico ADMET analysis predicted pharmacokinetics and organ‐specific toxicity. Fresh camel milk from the Brella and Marecha breeds was used. Liposomes were prepared via thin‐film hydration. SEM showed empty liposomes were spherical, while DTX‐loaded liposomes were rectangular; FTIR confirmed successful encapsulation. Zeta analysis revealed particle size 836.6 nm, PDI 0.088, and zeta potential −18.7 mV. Encapsulation efficiency was 25%. In vitro release showed sustained DTX release. MTT assay on MDA‐MB‐231 cells at 80, 120, and 180 μg/mL showed dose‐ and time‐dependent cytotoxicity, with 60.2% inhibition at 180 μg/mL. Empty liposomes were non‐toxic. Liposomes were stable for 24 h at 4°C (Kousar et al. 2024). Krishnankutty et al. treated HCT 116 and MCF‐7 cells with camel milk. Proliferation, viability, and migration decreased significantly. Autophagy was induced, evidenced by LC3‐II accumulation, reduction of p62 and Atg 5‐12, and GFP‐LC3 puncta observed via confocal microscopy (Krishnankutty et al. 2018).

Hasson et al. evaluated lyophilized camel milk on BT‐474 and HEp‐2 cells compared with non‐cancer HCC1937 BL cells. BT‐474 proliferation was suppressed through intrinsic and extrinsic apoptosis, as indicated by caspase‐3 mRNA and activity and death receptor induction. Oxidative stress markers HO‐1 and ROS increased. The rise in caspase‐3 was blocked by actinomycin D, indicating RNA transcription dependence. Growth was significantly reduced in BT‐474 and HEp‐2 at 72 h and in BT‐474 alone at 24 h. Apoptosis was triggered via alternative pathways (Hasson et al. 2015).

#### 4.3.7 Anti‐Inflammatory Effect

Omega‐6 fatty acids, which are bad for you, increase inflammation in the body. Camel milk contains significant amounts of omega‐3 fatty acids, which naturally reduce inflammation (Alagawany et al. 2022). Camel milk contains vitamins A and B‐2, has a high level of vitamin C, and is abundant in zinc and magnesium, all of which have anti‐inflammatory properties against several viral disorders (Vincenzetti et al. 2022; Singh et al. 2023). These vitamins are helpful in reducing oxidative stress brought on by a hazardous substance. Magnesium is vital for the manufacture of glutathione, which protects cellular components from damage brought on by free radicals, peroxides, and heavy metals (Sharma et al. 2025). At the moment, magnesium significantly speeds up the antioxidant defense system (Mohamed et al. 2015).

Behrouz et al. evaluated the effects of camel milk on systemic oxidative stress and inflammation in 35 male Wistar rats with CS‐induced COPD. Rats were divided into control, CS‐exposed, CS + 4 mL/kg CM, CS + 8 mL/kg CM, and CS + 1 mg/kg dexamethasone groups. CS exposure increased WBC counts, TNF‐α, and MDA levels in serum and tissues, while decreasing CAT, SOD, and thiol levels. Treatment with camel milk or dexamethasone improved all measured variables, with high‐dose CM showing greater effects than dexamethasone (Behrouz et al. 2024). Bakhtiari et al. conducted a 2‐month double‐blind RCT in 60 children (> 6 years) with poorly controlled asthma. Participants received 200 mL of CM or a placebo daily. Fifty‐seven completed the study. CM significantly reduced the use of inhaled corticosteroids, short‐acting beta agonists, and long‐acting beta agonists. Changes in FEV1 were 21.89 ± 17.83 vs. 18.54 ± 14.89, and FEV1/FVC changes were 11.11 ± 8.33 vs. 8.11 ± 7.12 in CM vs. control (Bakhtiari et al. 2021). Ravaghi et al. performed a 3‐month RCT in 46 asthma patients. The intervention group received 250 mL of CM twice daily plus standard therapy, while the control group received standard therapy alone. Post‐treatment, FEV1 and FEV1% improved significantly in the CM group, and CAT scores also improved (Ravaghi et al. 2019).

#### 4.3.8 Iron Deficiency Anemia Management

High levels of iron found in camel milk help prevent iron deficiency anemia (Abdurahman and Gashu 2021). Red blood cells, which are crucial for the transport of oxygen and the creation of deoxyribonucleic acid, must include iron (Kuhn et al. 2017). Camel milk can considerably aid in preserving health and well‐being after childbirth, damage, or a time of malnutrition (Chikha and Faye 2025). Lactoferrin is an iron‐binding glycoprotein that is a small component of whey proteins (Niaz et al. 2019). It is also found in large concentrations in the breast secretions of nonlactating dairy animals. Its structure consists of two lobes, each of which can reversibly bind two atoms of iron (one for each lobe). One bicarbonate ion is synergistically linked to each lobe (Shimazaki 2000).

Abdurahman et al. assessed hemoglobin levels in 332 children aged 6–59 months from Somali pastoral communities, comparing 166 camel milk and 166 cow milk consumers. Among participants, 38.6% were underweight, 33.4% were stunted, 34.5% were wasted, and 77.4% were anemic. WASH conditions were poor, and only 0.6% had minimum dietary diversity. Camel milk consumers had higher mean hemoglobin than cow milk consumers, and cow milk consumers had higher odds of low hemoglobin. Camel milk improved hemoglobin but may not fully prevent anemia (Abdurahman and Gashu 2021). Muleta et al. conducted a cross‐sectional study with 388 children aged 24–59 months in Somali pastoral districts: 185 consumed camel milk and 203 consumed bovine milk. Anemia prevalence was 59.8%, with 42.7% in CaM and 75.4% in BM consumers. The odds of anemia were higher in BM consumers and in children with intestinal parasites. Older age and higher height‐for‐age z‐scores reduced anemia risk. Camel milk consumption was associated with lower anemia prevalence and may help reduce anemia in pastoralist children (Muleta, Hailu, and Belachew 2021; Muleta, Hailu, Stoecker, et al. 2021).

#### 4.3.9 Viral Hepatitis Management

Camel milk contains fats that calm the liver and help treat chronic liver disease adjuvantly (Shakeel et al. 2022). Improved liver function may be assisted by camel milk's high ascorbic acid content (Korish and Arafah 2013). Both hepatitis B and hepatitis C may be cured with camel milk (El‐Fakharany et al. 2017). Hepatitis C and B viruses can be inhibited by the lactoferrin found in camel milk, which stops them from replicating in cells (Almahdy et al. 2011). Camel milk may treat hepatitis B because it boosts the immune system and prevents the virus's DNA from replicating (Khan et al. 2021).

Redwan et al. evaluated camel milk in HCV‐infected patients, administering 250 mL/day for 4 months. ALT decreased in 88% of patients and AST in all patients. Viral load decreased in 13 of 17 patients (76.47%), with one patient achieving undetectable RNA. IgG1 anti‐HCV antibodies significantly decreased in 70%–76% of patients, indicating a shift toward Th1 immunity. Treatment was ineffective in 23.53% of patients with no viral load reduction, but overall, camel milk was safe and improved liver function and immunity (Redwan et al. 2017). Rusu et al. compared a normoglucidic low‐calorie diet (NGLCD) versus a low‐fat diet (LFD) in 120 overweight or obese CHC patients over 12 months. At 6 months, NGLCD led to greater weight loss, though at 12 months, weight loss was similar between groups. Both diets improved fasting glucose, insulin, HOMA‐IR, AST, ALT, GGT, AST/ALT ratio, Forns fibrosis index, steatosis, and lipid profiles, demonstrating effectiveness in reversing insulin resistance, obesity, steatosis, and fibrosis (Rusu et al. 2013).

#### 4.3.10 Antiparasitic Effects

*Schistosoma mansoni* can be eradicated by the anti‐parasitic properties of lactoferrin found in camel milk (Reyes‐López et al. 2022). Colostrum and mature camel milk are given to the patient to encourage a specific immune response that protects against *Schistosoma mansoni* (Almasri et al. 2024). The body's level of glutathione‐transferase (GST), which is improved as a result of the immune‐protective phenomenon, detoxifies the body more effectively (Almasri et al. 2024). This led to the announcement that camel milk that is mature and colostral camel milk has a preventive effect against schistosomiasis infection.

Maghraby et al. evaluated the anti‐schistosomal effects of colostral and mature camel milk in *Schistosoma mansoni* –infected mice. Six weeks post‐infection, protection rates were 12.81% for colostral milk and 31.60% for mature milk. Serum GST increased over time in both colostral and mature milk groups compared with controls, whereas ALT and AST showed only slight changes. Camel milk (200 μL/day) also induced IgG against SWAP (0.31 ± 0.1 for colostrum; 0.34 ± 0.1 for mature milk) versus controls (0.2 ± 0.04), with levels remaining stable over time (Maghraby et al. 2005). These findings indicate that both colostral and mature camel milk exert immunomodulatory effects, enhancing GST and IgG and providing partial protection against schistosomiasis.

#### 4.3.11 Antihypertensive Effects

Angiotensin‐converting enzyme (ACE), a dipeptidyl‐carboxypeptidase, catalyzes the transformation of the inert angiotensin I peptide into the powerful vasoconstrictor angiotensin II peptide (Bakhle 2020). The increase in salt levels caused by angiotensin II increases blood pressure (Berecek et al. 2018). The ACE‐inhibitory activity of camel milk protein hydrolysates has been examined, and it has been found that camel whole casein and camel β‐CN have increased ACE inhibitory activities after enzymatic hydrolysis of camel milk proteins (Alhaj et al. 2024). After hydrolysis with pepsin alone and after hydrolysis with pepsin followed by trypsinolysis and chymotrypsinolysis, camel whole casein and β‐CN showed strong ACE‐inhibitory actions (Jiang et al. 2007). Reversed‐phase HPLC (RP‐HPLC) can be used to identify the ACE inhibitory peptides, and triple mass spectrometry can be used to measure their concentration (Khakhariya et al. 2023).

Alshuniaber et al. evaluated the antihypertensive effect of camel milk hydrolysate (CMH) in 36 Wistar male rats with 10% fructose‐induced hypertension. Rats were orally treated with 800 mg/kg or 1200 mg/kg CMH for 21 days. The 1200 mg/kg CMH group showed significant reductions in systolic and diastolic blood pressure after 4–8 h, comparable to nifedipine. ACE activity decreased in CMH‐treated groups, correlating with blood pressure reduction. Fructose‐fed rats had elevated glucose, insulin, triglycerides, and total cholesterol, which were lowered toward baseline in NIF, HM1200, and HM800 groups (Alshuniaber et al. 2021). Alshuniaber et al. compared camel milk protein hydrolysate (CMH) and intact camel milk (ICM) in 48 high‐fructose‐fed rats over 21 weeks. Both treatments reduced fasting glucose, insulin, serum and hepatic cholesterol and triglycerides, ALT, AST, angiotensin II, ACE, endothelin‐1, uric acid, hepatic fat deposition, and hepatocyte damage. CMH enhanced AMPK activity, increased PPARα mRNA, and downregulated fructokinase C, SREBP1, SREBP2, fatty acid synthase, and HMG‐CoA reductase. Both treatments lowered systolic and diastolic blood pressure, with CMH showing greater effects (Alshuniaber et al. 2022).

#### 4.3.12 Improve Food and Milk Allergies

Proteins in camel milk have a crucial role in avoiding and treating food allergies (Arain et al. 2023). Camel milk lacks β‐lactoglobulin and has a different β‐casein (Kula 2016; Muthukumaran et al. 2023). It contains immunoglobulins identical to those found in mother's milk, which lessen allergic reactions in children and improve their ability to respond to foods in the future. Lactose intolerance is classified as being caused by a lack of lactase, the enzyme required to break down lactose found in dairy products (Muthukumaran et al. 2023). Bloating, diarrhea, and gastrointestinal difficulties after consuming dairy products are symptoms of lactose intolerance (Muthukumaran et al. 2023). Camel milk has very little of the lactose and casein that cause lactose intolerance (E. S. I. El‐Agamy 2006). The two most frequently associated allergenic proteins in cow milk, A1 casein and lactoglobulin, are absent from camel milk (Elagamy 2016). A lactase‐producing enzyme that the body secretes enables the body to digest sugar (Zuhri and Wilda 2024; Acharya et al. 2025). This enzyme is absent in some people, which causes insufficient breakdown of lactose sugar. Only a small amount of lactose, which can be easily digested by those with lactose intolerance, is present in camel milk (Cardoso et al. 2010). Additionally, there is a minor amount of A1 casein, which is indigestible to people with lactose intolerance or dairy product allergies (Konuspayeva and Faye 2021).

Navarrete‐Rodríguez et al. evaluated the safety of camel's milk in children (1–18 years) with CMPA in a crossover trial. CMPA affects 0.6%–0.9% of the population and requires complete avoidance of cow's milk protein. Participants confirmed by DBPCFCs received either camel's milk or an amino acid formula for 2 weeks, followed by a 6‐week washout and crossover. No adverse effects were observed, demonstrating that camel's milk is safe, tolerable, and a palatable alternative (Navarrete‐Rodríguez et al. 2018).

#### 4.3.13 Limitations of Included Studies on the Therapeutic Benefits of Camel Milk

However, most studies on camel milk have important limitations that affect interpretation and generalizability. Many clinical trials and intervention studies, such as those by Wang et al. (2009), Sboui et al. (2022), Zheng et al. (2021), Al‐Ayadhi et al. (2022), and Bashir and Al‐Ayadhi (2014), had small sample sizes, short durations, and, in some cases, lacked blinding or proper controls, which may introduce bias. Meta‐analyses, such as Kandeel et al. (2024), were limited by heterogeneity and the small number of included trials, producing nonsignificant pooled effects for some outcomes. Several studies, including Shaban et al. (2023), Hussein and Muhialdin (2021), Abbes et al. (2021), Kousar et al. (2024), Krishnankutty et al. (2018), and Hasson et al. (2015), were conducted in vitro or in animal models, limiting applicability to humans. Observational studies like Abdurahman and Gashu (2021), Muleta, Hailu, and Belachew (2021), and Muleta, Hailu, Stoecker, et al. (2021) were prone to confounding and could not establish causality. Case reports, such as those of Rosenheck et al. (2012), cannot determine effectiveness. Some intervention studies, including Tahir et al. (2022), Redwan et al. (2017), and Masika et al. (2025), involved very small participant numbers or short follow‐ups, limiting statistical power and long‐term conclusions. Finally, studies in animal models or using hydrolysates (Alshuniaber et al. 2021, 2022; Maghraby et al. 2005) provide mechanistic insight but may not fully translate to clinical efficacy in humans.

## 5 Zoonotic Risks Associated With Camel Milk Consumption

Camel milk is nutritionally valuable but may carry zoonotic pathogens if consumed raw or unpasteurized (Khalafalla 2023) (Table 9). Zoonosis refers to the natural transmission of infectious agents from vertebrate animals to humans (Khalafalla 2023). Common bacterial contaminants include Salmonella, Brucella, Campylobacter, *Staphylococcus aureus* , and *Clostridium perfringens* , which can lead to gastrointestinal infections, brucellosis, and other illnesses (Ali and Alsayeqh 2022). Contamination often occurs during camel milking, handling, or storage (Odongo et al. 2016).

**TABLE 9:** Bacterial risks in raw camel milk from the included studies.

Bacterial pathogen	References	Location	Study design	Sample size and source	Findings/prevalence	Health risks/illnesses	Additional notes
Salmonella spp.	Elhaj and AlSobeai (2018)	Sajer, Saudi Arabia	Cross‐sectional observational	30 raw camel milk samples from farms	Detected Salmonella along with Bacillus spp., *E. coli* , *Staphylococcus epidermidis* , Pantoea spp., and Klebsiella spp.	Diarrhea, vomiting, abdominal cramps, foodborne infections, TB, brucellosis, meningitis	Raw milk is consumed directly without pasteurization; general bacterial contamination is high.
*Salmonella enterica*	Matofari et al. (2007)	Kenya	Cross‐sectional	196 samples: individual milk, bulk milk, feces, soil, water	43% of samples positive; 31% confirmed *S. enterica*	Gastrointestinal infections, salmonellosis	Contamination mainly due to post‐harvest handling; the presence of Typhi indicates human fecal contamination
Brucella spp.	Ambaw et al. (2025)	Dire District, Ethiopia	Observational	Camel farmers are consuming unpasteurized milk	Higher risk of human brucellosis (OR: 2.75, 95% CI: 1.51–5.21)	Brucellosis: fever, joint pain, seropositive	Households with infected camels increased risk; this highlights the need for pasteurization.
*Brucella melitensis*	Shimol et al. (2012)	Southern Israel	Outbreak investigation	15 Bedouin family members are consuming unpasteurized milk	Patients infected; camel milk tested positive	Human brucellosis	Demonstrates familial outbreaks from raw camel milk
	Bardenstein et al. (2021)	Israel	Observational outbreak	WGS Commercially sold camel milk	Linked infections in humans to camel milk	Human brucellosis	Whole‐genome sequencing traced infection to milk supply; risk in unregulated trade.
*Campylobacter jejuni* / *C. coli*	Mohammed et al. (2022)	Qatar	Quantitative risk assessment	Repeated cross‐sectional surveys	Healthy women: 0.5%–2.4% risk; healthy men: 1.3%–3.0%; higher risk in immunocompromised	Gastrointestinal illness, Campylobacteriosis	Consumption of fresh, unpasteurized milk; QRA used to estimate risk
*Staphylococcus aureus*	Tasse et al. (2022)	Fedis, Ethiopia	Cross‐sectional	Raw camel milk samples (38.5% contaminated)	38.3% exceeded safe bacterial limits	Bacterial infection, foodborne intoxication	Contamination is higher in market samples, influenced by camel age, parity, lactation stage, and milk source.
	Serda et al. (2018)	Ethiopia	Cross‐sectional	384 raw camel milk samples from households, collection centers, selling sites	11.45% overall prevalence; mean counts: 4.2 × 10^4^ CFU/mL	Bacterial infection, foodborne intoxication	Contamination occurs from udder/handlers; potential for staphylococcal toxin production resistant to heat
Clostridium spp./ *C. perfringens*	Aliwa and Mulwa (2019)	Isiolo County, Kenya	Cross‐sectional	308 raw camel milk samples from 15 herds	48.05% positive for Clostridium spp.; 19.1% *C. perfringens* ; multi‐drug resistant	Food poisoning, gastrointestinal illness, tetracycline, gentamicin	High antibiotic resistance; resistance to ampicillin, sulfamethoxazole, cotrimoxazole, streptomycin, chloramphenicol, and kanamycin

### 5.1 Salmonella

Salmonella is a genus of Gram‐negative, rod‐shaped bacteria belonging to the family Enterobacteriaceae (Oludairo et al. 2022). Many species and serovars can cause gastrointestinal infections in humans and animals (Oludairo et al. 2022). Salmonella contamination in camel milk occurs primarily through post‐milking handling, contaminated water, and poor hygiene rather than direct infection of camels (Amenu et al. 2019). Elhaj et al. conducted a cross‐sectional observational study on the bacterial contamination of raw camel milk, which is commonly consumed directly after milking without pasteurization. Thirty milk samples were aseptically collected from farms in the Sajer region and analyzed in the laboratory for aerobic bacteria. Pathogenic microorganisms detected included Bacillus spp., *Staphylococcus epidermidis* , *Escherichia coli* , Salmonella spp., Pantoea spp., and Klebsiella spp. (Elhaj and AlSobeai 2018). These bacteria pose significant health risks to humans, and their presence in raw camel milk is associated with illnesses such as diarrhea, vomiting, abdominal cramps, foodborne infections, tuberculosis, brucellosis, and meningitis.

A cross‐sectional study in Kenya, conducted by Matofari et al., analyzed 196 samples, including milk from individual camels, bulk milk, feces, soil, and water, and found Salmonella species in 43% of samples, with 31% confirmed as *Salmonella enterica* . Contamination was present across all sample types and was primarily linked to postharvest handling rather than camel infection. The detection of serovar Typhi, a human‐adapted pathogen, indicates human fecal contamination (Matofari et al. 2007). These findings show that consuming raw camel milk may cause salmonellosis and other gastrointestinal infections.

### 5.2 Brucellosis

Brucella are small, Gram‐negative, facultative intracellular coccobacilli that cause brucellosis, a zoonotic disease affecting various mammals, including camels, cattle, sheep, and goats (Ghssein et al. 2025). Camel milk is a recognized vehicle for *Brucella melitensis* and other Brucella species (Adel 2022). Human brucellosis is often contracted through consumption of unpasteurized milk (Garcell et al. 2016).

In Dire District, Ethiopia, Ambaw et al. found that camel farmers who consumed unpasteurized camel milk had a significantly higher risk of Brucella infection. The study also indicated that households with Brucella‐positive camels contributed to this risk, highlighting a direct link between contaminated milk and human infection (Ambaw et al. 2025). Shimol et al. reported an observational outbreak of human brucellosis linked to the consumption of unpasteurized camel milk among 15 Bedouin family members in southern Israel. Milk samples from the camel tested positive for *Brucella melitensis* , and the patients developed fever, joint pain, and positive serology or blood cultures (Shimol et al. 2012). This study highlights that unpasteurized camel milk can transmit zoonotic diseases, such as brucellosis, posing a risk in both sporadic and familial outbreaks.

Bardenstein et al. reported an observational outbreak investigation in Israel linking human brucellosis to commercially sold camel milk contaminated with *Brucella melitensis* . Using whole‐genome sequencing, the researchers traced the infections in patients directly to the camel milk supply and the associated unregulated livestock trade (Bardenstein et al. 2021). This study highlights the potential zoonotic risk of consuming unpasteurized or unregulated camel milk, demonstrating that commercially distributed camel milk can serve as a source of *Brucella melitensis* transmission to humans.

Garcell et al. conducted a descriptive observational case series reporting a human brucellosis outbreak in 14 family members from a rural area in Qatar. Clinical, epidemiological, and laboratory data were collected for all 14 confirmed cases and 12 nonconfirmed relatives. All patients experienced fever for up to 14 days; other symptoms included arthralgia (6/14), weakness (4/14), headache (4/14), diarrhea (2/14), and abdominal pain (2/14). Elevated transaminases were observed, and blood cultures with serology identified mixed infection with *Brucella abortus* and *Brucella melitensis* . The source of infection was milk from an infected camel (Garcell et al. 2016).

### 5.3 *Campylobacter jejuni*

Campylobacter spp. are Gram‐negative, spiral‐shaped bacteria commonly associated with foodborne gastroenteritis (Ammar et al. 2021). *C. jejuni* is the most prevalent cause of bacterial diarrhea worldwide (Silva et al. 2011). Camel milk can occasionally be contaminated with Campylobacter spp., posing a risk for gastroenteritis (Mohammed et al. 2022). Mohammed et al. conducted a quantitative risk assessment (QRA) study to evaluate the risk of illness from consuming fresh camel milk contaminated with *Campylobacter jejuni* and *C. coli* in Qatar. Data were collected through repeated cross‐sectional surveys in camel milk and human populations. Healthy women consuming contaminated milk had a 0.5%–2.4% risk of illness, and healthy men had a 1.3%–3.0% risk. Immunocompromised females and males faced three‐ and fivefold higher risks, respectively (Mohammed et al. 2022).

### 5.4 *Staphylococcus aureus*

*Staphylococcus aureus* is a Gram‐positive, facultatively anaerobic coccus known for producing enterotoxins that can cause food poisoning (Fayisa and Tuli 2023). It can colonize human and animal skin and mucosa. Raw camel milk can be contaminated with *S. aureus* through direct contact with the animal's udder or from handlers (Serda et al. 2018). Serda et al. conducted a cross‐sectional study on 384 raw camel milk samples from households, primary collection centers, and selling sites to determine *Staphylococcus aureus* prevalence and associated risk factors. The overall prevalence was 11.45% (44/384): 7.03% (9/128) at households, 11.71% (15/128) at collection centers, and 15% (20/128) at selling sites. Mean counts were 4.2 × 10^4^ CFU/mL overall, with 8.9 × 10^2^, 9.9 × 10^3^, and 1.1 × 10^5^ CFU/mL at households, collection centers, and selling sites, respectively (Serda et al. 2018).

A cross‐sectional study by Tasse et al. in Fedis, Eastern Hararghe, Ethiopia, found that 38.5% of raw camel milk samples were contaminated with *Staphylococcus aureus* , and 38.3% of these exceeded safe bacterial limits. Contamination was higher in market samples than in household samples and was influenced by factors such as camel age, parity, lactation stage, and milk source (Tasse et al. 2022). These findings indicate that consuming raw or improperly handled camel milk can result in bacterial infections and intoxication.

### 5.5 Clostridium

Clostridium species are Gram‐positive, anaerobic, spore‐forming bacteria (Stevens et al. 2015). *C. perfringens* is notorious for causing food poisoning and necrotic enteritis (Fu et al. 2022). Camel milk may harbor Clostridium spores, especially in unsanitary or improperly stored conditions (Shalini and Gurunathan 2025). In a cross‐sectional study, Aliwa et al. collected 308 raw camel milk samples from 15 herds in Isiolo County, Kenya. Of these, 48.05% tested positive for Clostridium species, and 19.1% were confirmed as *C. perfringens* . Antibiotic resistance among the isolates was high, with resistance observed to ampicillin (61.0%), sulfamethoxazole (47.5%), cotrimoxazole (45.8%), streptomycin (44.1%), chloramphenicol (42.4%), kanamycin (40.7%), tetracycline (37.3%), and gentamicin (35.6%) (Aliwa and Mulwa 2019). These findings indicate that raw camel milk may harbor multidrug‐resistant *C. perfringens* .

### 5.6 Limitations of Studies on Zoonotic Risks of Camel Milk

However, several studies on raw camel milk contamination and zoonotic risks have methodological limitations that should be considered. Elhaj and AlSobeai (2018) used a small sample size and limited geographic coverage, while Matofari et al. (2007) relied on convenience sampling and focused on postharvest contamination rather than camel‐specific sources. Ambaw et al. (2025) and Shimol et al. (2012) were observational with small populations, limiting generalizability and control of confounding factors. Bardenstein et al. (2021) also faced limited applicability due to outbreak‐specific data and small sample sizes. Garcell et al. (2016) reported a case series without a control group, restricting external validity. Mohammed et al. (2022) conducted a quantitative risk assessment that relied on model assumptions and self‐reported exposure, which may not fully reflect real‐world variability. Cross‐sectional studies such as Serda et al. (2018), Tasse et al. (2022), and Aliwa and Mulwa (2019) were limited by convenience sampling, regional constraints, and lack of long‐term follow‐up, and some did not fully control for contamination factors or explore mechanisms of antibiotic resistance.

## 6 Minimizing Zoonotic Risks Associated With Camel Milk

Zoonotic diseases often arise not only from infected animals but also from post‐harvest handling, poor hygiene during milking, improper storage, and unregulated distribution (Seyoum et al. 2024). For instance, the detection of human‐adapted pathogens such as *Salmonella Typhi* indicates fecal contamination during handling rather than inherent infection in camels (Duchêne et al. 2020). Furthermore, multidrug‐resistant strains of *Clostridium perfringens* highlight the risk of resistant infections from unpasteurized milk (AlJindan et al. 2023). Pasteurization of camel milk before consumption is the most effective method to eliminate pathogenic bacteria (Dhahir et al. 2021). Ensuring proper hygiene during milking, using sterilized containers, and maintaining cold chain storage can significantly reduce microbial contamination (Konuspayeva and Faye 2021). Additionally, educating camel herders, milk handlers, and consumers about zoonotic risks and safe milk handling practices is crucial (Konuspayeva and Faye 2021). Regulatory oversight of commercially sold camel milk, including routine microbiological testing, labeling, and adherence to safety standards, further minimizes public health hazards (Yang et al. 2025). Implementing these measures collectively ensures that the nutritional and therapeutic benefits of camel milk can be safely harnessed while reducing the risk of foodborne illnesses (Haldar et al. 2022).

Special precautions should be taken for vulnerable populations, including children, pregnant women, the elderly, and immunocompromised individuals (Doherty et al. 2016). For these groups, only pasteurized or properly treated camel milk should be consumed, and extra care must be given to hygiene, storage, and handling to prevent exposure to pathogenic microorganisms. Implementing these measures ensures that the nutritional and therapeutic benefits of camel milk can be safely realized while minimizing the risk of foodborne illnesses (Kim et al. 2021).

## 7 Limitations

This review has several limitations. As a nonsystematic review, it lacks meta‐analytic quantification and may be subject to selection bias. The inclusion of only English‐language studies was potentially excluding relevant findings. Variations in camel breeds, milk processing, study designs, and populations limit direct comparability. Moreover, most available data derive from preclinical models, reducing generalizability to humans. Small clinical sample sizes, short intervention durations, and limited reporting of confounders, dosages, or long‐term safety further restrict the strength of the conclusions.

## 8 Future Directions

Future research should focus on well‐designed, large‐scale clinical trials to validate the therapeutic and physiological benefits of camel milk across diverse populations. Standardization of composition, processing methods, and dosage is essential to ensure reproducibility and efficacy. Long‐term safety studies, particularly regarding raw milk consumption, are required to assess potential zoonotic or prion‐related risks. Further, mechanistic studies should further investigate the roles of bioactive compounds in immune modulation, metabolic regulation, and disease prevention. Comparative studies across breeds and regions, along with the development of functional foods and nutraceutical applications, may enhance its translational and clinical potential.

## 9 Conclusion

This review highlights camel milk as a nutrient‐dense functional food with diverse therapeutic, physiological, and nutritional benefits. Preclinical and clinical evidence support its roles in metabolic regulation, immune modulation, antimicrobial activity, and growth promotion, with a generally favorable safety profile. However, unpasteurized camel milk may pose a risk of zoonotic infection and should be carefully handled and properly processed to minimize microbial and allergenic risks. Bioactive compounds such as whey proteins, casein derivatives, exosomes, and probiotics likely mediate therapeutic effects, offering potential for disease prevention and complementary therapies. By consolidating current knowledge, the review underscores camel milk's emerging role in modern healthcare. Further standardized clinical and mechanistic studies are warranted to fully unlock its therapeutic and nutritional potential while ensuring safety.

## Author Contributions

**Gudisa Bereda:** conceptualization, administration, supervision, methodology, writing – original draft, writing – review and editing. The author has read and approved the final version of the manuscript. **Subasini Uthirapathy; Javed Ahamad:** supervision, reading, editing, and contribution to methodology, writing – original draft, writing – review and editing. All authors have read and approved the final version of the manuscript.

## Funding

The authors have nothing to report.

## Conflicts of Interest

The authors declare no conflicts of interest.

## Data Availability

Data sharing not applicable to this article as no datasets were generated or analyzed during the current study.
